# Betel nut sucking is the earliest recorded evidence for the habitual use of a neuroactive plant drug

**DOI:** 10.1126/sciadv.aei1901

**Published:** 2026-09-09

**Authors:** Adam Brumm, Fakhri, Melandri Vlok, Roger L. Papke, Carney D. Matheson, Budianto Hakim, Clare Stokes, Basran Burhan, Suryatman, Andi Muhammad Saiful, Michelle C. Langley, Pratiwi Yuwono, Delta Bayu Murti, Sofwan Noerwidi, Marlon N. R. Ririmasse

**Affiliations:** ^1^ Australian Research Centre for Human Evolution, Griffith University, Brisbane, Queensland, Australia.; ^2^ School of Environment and Science, Griffith University, Brisbane, Queensland, Australia.; ^3^ Pusat Riset Arkeometri, Badan Riset dan Inovasi Nasional (BRIN), Jakarta, Indonesia.; ^4^ Pusat Kolaborasi Riset Arkeologi Sulawesi (BRIN-Universitas Hasanuddin), Makassar, Indonesia.; ^5^ School of Dentistry and Medical Sciences, Charles Sturt University, Orange, New South Wales, Australia.; ^6^ Department of Pharmacology and Therapeutics, College of Medicine, University of Florida, Gainesville, FL, USA.; ^7^ Pusat Riset Arkeologi Prasejarah dan Sejarah, BRIN, Jakarta, Indonesia.; ^8^ Departemen Arkeologi, Universitas Hasanuddin, Makassar, Indonesia.; ^9^ Southern Cross University, Lismore, New South Wales, Australia.; ^10^ Departmen Antropologi Ragawi, Universitas Airlangga, Surabaya, Indonesia.; ^11^ Pusat Riset Arkeologi Lingkungan, Maritim, dan Budaya Berkelanjutan, Organisasi Riset Arkeologi, Bahasa, dan Sastra, BRIN, Jakarta, Indonesia.

## Abstract

The modern custom of chewing betel nuts is practiced by around 1 in 10 people worldwide, yet the earliest beginnings of this widespread drug use habit are unknown. We report rare skeletal remains of two preagricultural foragers from Sulawesi (Indonesia), both displaying a dental pathology that we establish was caused by a hitherto unknown precursor to betel chewing: habitual sucking of whole betel nuts. Previous reports suggested that the main neuroactive alkaloid in betel nut, arecoline, is released by masticating the processed nut with slaked lime. However, our experiments demonstrate that simply keeping intact betel nuts in the oral cavity for prolonged periods releases arecoline. Biomolecular analysis of the ancient human teeth is also consistent with this early form of ingesting betel nut. With the earliest forager dating to 25 to 16 thousand years ago, our findings suggest the use of betel nut as a drug has more ancient origins than previously supposed.

## INTRODUCTION

Humans are prolific consumers of substances that alter our brain chemistry, and there is no known cultural group that does not use narcotics, stimulants, hallucinogens, or “drugs” of some kind (*1*–*3*). Despite this, very little is known about the origins of our oldest psychoactive agents. Based on current archaeological evidence, the most ancient substances used by humans to induce altered states of consciousness, whether for religious or medical purposes or simple recreation, are plant-based drugs (*1*–*3*). In order of antiquity, these are (*3*): beer, first made from barley (*Hordeum* spp.) some 13 thousand years ago (ka) in Israel; San Pedro cactus (*Trichocereus* spp.) and Mescalbean (*Sophora secundiflora*) (10.4 ka in Peru and Texas, respectively); coca (*Erythroxylum* spp.) (8 ka in Peru); and psilocybian mushrooms (8 ka in the Sahara). In all cases, the earliest attested evidence for these plants in the archaeological record probably does not reflect the true antiquity of associated drug practices (*1*–*3*). Moreover, almost nothing is known about the exact origins of these and other early plant drugs, that is, how past people invented or discovered them in the first place (*1*–*3*). It is often reasoned that humans acquired knowledge of the properties of psychotropic flora through a process of trial and error (e.g., accidental ingestion) and by observing animal behavior (*2*). Such ideas are difficult if not impossible to test. Another commonly held assumption is that most if not all drug plants can be traced to the emergence of the first sedentary farming societies (the so-called “Neolithic Revolution”). This seems unlikely, however, given that most traditional foraging peoples have acquired an extensive understanding of botanical pharmacology, and some groups do intentionally consume psychotropic plants within long-standing cultural practices (*2*). Given these gaps in our knowledge, any finding that yields empirical insight into the earliest human drug taking, especially among preagricultural foragers, is important.

### Betel nut

**Fig. 1.**
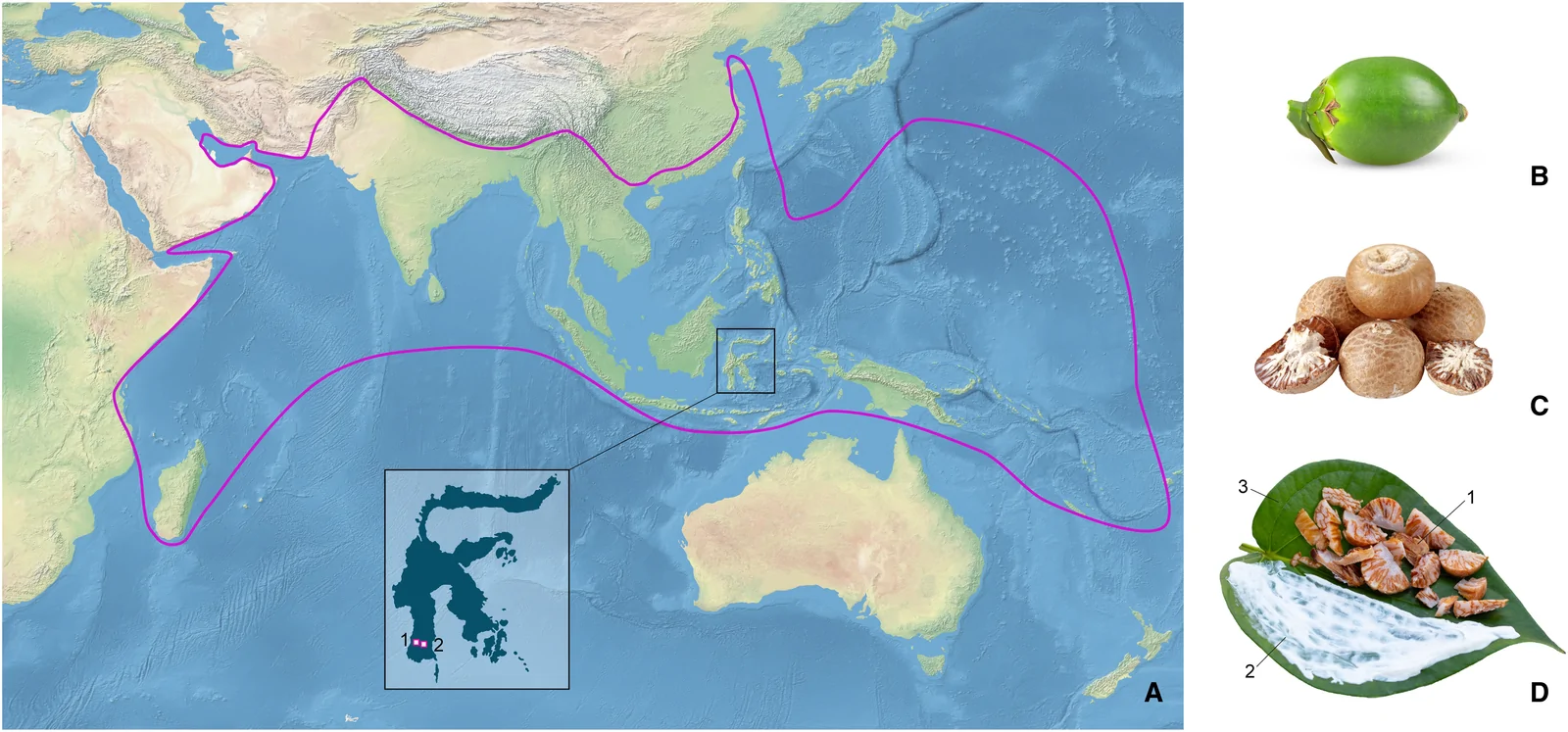
Historical distribution of betel chewing and elements of the betel quid. (**A**) The purple line denotes the region within which betel quid usage is common or had been until recent times. The Indonesian island of Sulawesi is shown inset, with the locations of the two archaeological sites in the island’s southwestern peninsula indicated (1, Leang Bulu Bettue; 2, Leang Cappalombo 1). (**B** to **D**) basic constituents of a betel quid; (B) a young *A. catechu* fruit (the betel “nut”) with the husk and pedicel intact; (C) dehusked kernels of the areca fruit; (D) elements of a prepared quid, the three key ingredients comprising: (1) sliced pieces of betel nut; (2) slaked lime paste; and (3) a leaf of the Betel vine (*Piper betle*). Map source: Made with Natural Earth, free vector, and raster map data @naturalearthdata.com. Source of betel distribution (*4*). Images in (B) to (D) licensed from Shutterstock.

A psychoactive plant source that is frequently listed among the world’s oldest known drugs (*1*–*3*) and is regarded by some authorities as the oldest is so-called betel nut. Also commonly known as betel, areca, *supari*, *pinang*, or *paan*, depending on the region, betel nut comprises the drupe fruit of various *Areca* spp. palms (*4*–*8*). The cultivated variant *Areca catechu* L. is the most widely used taxon (*4*–*8*). Although poorly known in western societies, betel nut is a cholinergic stimulant widely used throughout tropical Asia and the Pacific (*4*–*8*); it is reputed to be the world’s fourth most popular psychoactive substance after alcohol, caffeine, and nicotine (*6*). The consumption of betel quid as both recreational drug and traditional medicine is a culturally diverse custom with a geographic distribution extending from the South Pacific islands to the coast of eastern Africa, a vast area that incorporates the entire Indian subcontinent (India–Pakistan–Sri Lanka), Southeast Asia, most of Melanesia and Micronesia, and parts of coastal southern China (Fig. 1) (*4*). Particularly important for social bonding, betel quid usage is deeply embedded in the greeting customs and communal ceremonies (e.g., wedding rituals) of many groups throughout the region (*4*).

The drug is most typically self-administered by masticating a prepared quid composed of a processed areca seed, slaked lime powder or paste derived from pulverized shells (calcium hydroxide) or limestone (calcium carbonate), and the leaf of the Betel vine (*Piper betle* L.) (Fig. 1) (*4*–*7*). Betel quids are chewed in the buccal cheek cavity, with the blood-rich tissue in this part of the mouth allowing the plant compounds to enter the bloodstream more rapidly (*1*) [while also elevating the risk of oral cancer; (*9*)]. Slaked lime is an essential ingredient in the quid, as it has the effect of converting betel alkaloids—including what has been suggested to be the principal neuroactive compound, arecoline, a muscarinic agonist (*6*, *7*)—into free base forms, thereby increasing their bioavailability. Reported parasympathetic effects of betel nut ingestion include a heightened sense of alertness, mild euphoria, and increased stamina and capacity for work (*4*–*7*).

The practice of betel chewing has a long cultural history, with a written record predating the common era in India and China (*4*, *7*). Archaeological evidence for this custom, however, is not abundant, traditionally consisting of carbonized areca seed fragments and “betel stains” on human teeth (see the Supplementary Materials) (*8*, *10*). Concerning the latter, the addition of slaked lime to a betel quid alkalizes the oral cavity and is responsible for producing a bright red colorant that darkens over time to form the conspicuous reddish-brown stain on users’ teeth, due to the formation of secondary (oxidative) reaction products (*11*). However, long-standing claims (*8*) for the extreme antiquity of betel-stained dentitions at some archaeological sites in Southeast Asia (up to 13 ka in Thailand) suffer from poor dating resolution and/or are as-yet untested (*8*) (see the Supplementary Materials). Cosmetically dyed teeth (*12*) have probably also been misinterpreted as evidence for betel chewing in some instances (*8*).

Up until now, the earliest securely dated and relatively unambiguous indication of betel nut consumption consisted of chemical traces of arecoline found in the dental calculus of an individual from Bronze Age Thailand (∼3.5 to 3 ka) (*13*). It is highly likely, however, that prehistoric seafarers had translocated *A. catechu* to mainland Southeast Asia from its source of origin somewhere in the Indo-Malay archipelago (*8*). The most commonly accepted idea is that betel quid usage arose in the latter region following the arrival of Austronesian-speaking groups 4 ka at the earliest (*14*). Under this scenario, the descendants of these Neolithic farmers then introduced the betel chewing custom to more far-flung areas either directly or indirectly through a long history of migration and diffusion (*8*, *14*).

### Dental wear in the teeth of pre-Neolithic foragers from Sulawesi

We report here skeletal remains of two adult foragers from preagricultural Sulawesi, both of which exhibit a form of dental pathology (rounded, hollowed-out tooth grooves) that has not previously been recognized and formally described in the literature.

**Fig. 2.**
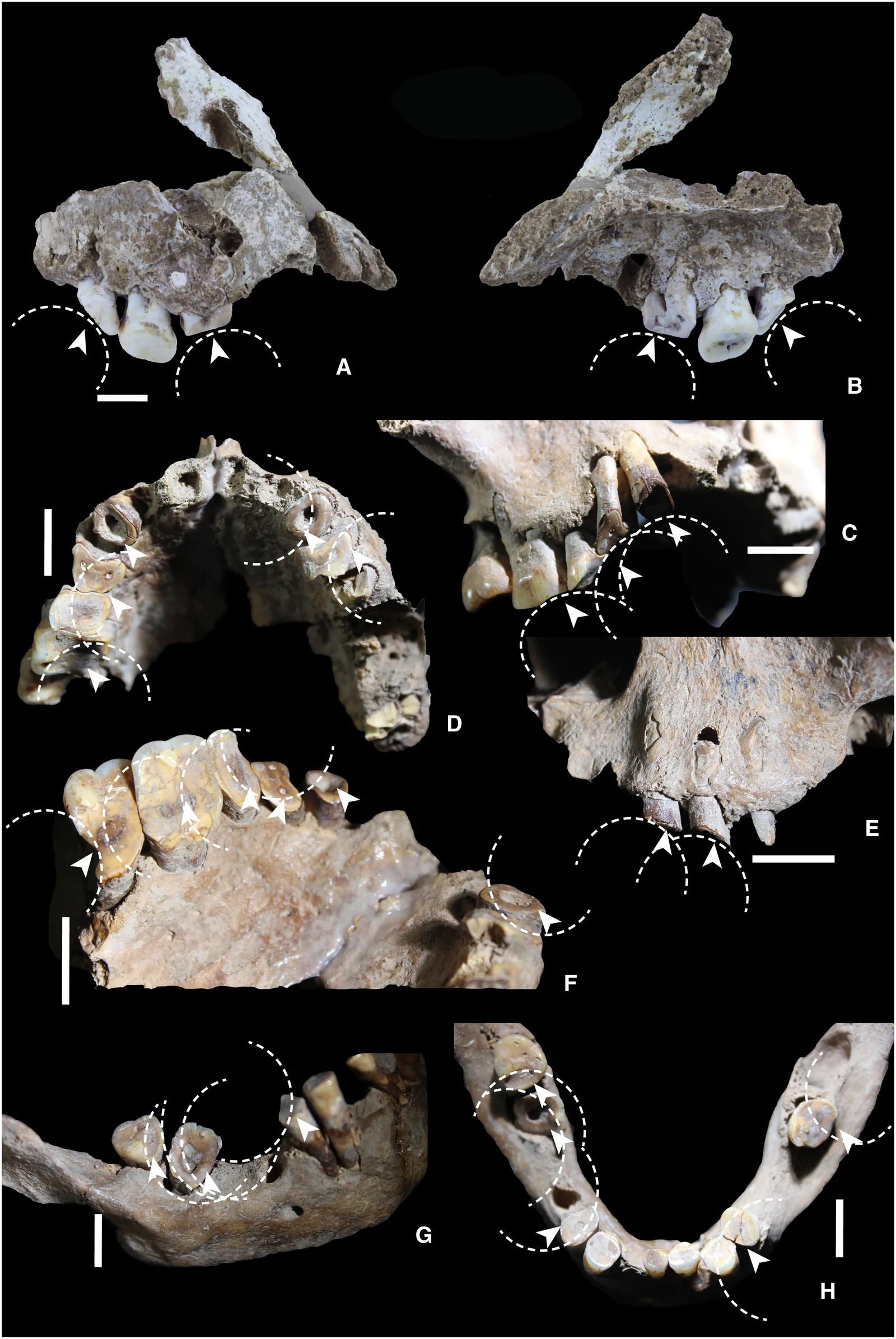
Drug use-related dental wear in early Sulawesi foragers. (**A** and **B**) Right maxilla fragment with three molars (M^1^ to M^3^) from a Late Pleistocene individual (Maros-LBB-1a); (A) right lateral view; (B) left lateral view. (**C** to **H**) Maxillary [(C) to (F)] and mandibular [(G) to (H)] dentition of a Middle Holocene individual (CPL-R2a). Both are middle to old adults. Circumferential wear facets are indicated by arrows and dotted half circles. Scale bars, 10 mm.

Skeletal elements attributed to the first forager (denoted Maros-LBB-1a) were excavated from Late Pleistocene deposits at Leang Bulu Bettue (Fig. 2 and fig. S1). Maros-LBB-1a is dated by multiple methods to 25 to 16 ka (*15*, *16*) (see Materials and Methods). Remains of the second individual (CPL-R2a) were excavated from a Holocene site, Leang Cappalombo 1, some 33 km to the east (Fig. 2 and figs. S2 to S4) (*17*). This second, much more complete, individual is associated with the Toalean technocomplex (∼8 to 1.5 ka), a localized foraging culture with deep pre-Neolithic roots in South Sulawesi (*18*, *19*). An age of 7.6 to 6.3 thousand calibrated radiocarbon years before the present (kyr cal B.P.) is inferred for CPL-R2a (Materials and Methods).

Skeletal remains of preagricultural foragers are extremely uncommon in south Sulawesi. Maros-LBB-1a is the only human skeletal element presently known from the Pleistocene record of this large (∼174,000 km^2^) island (*15*, *16*). Similarly, despite well over a century of archaeological research into the Toalean technocomplex (*18*), very few skeletal remains of these foragers have been recovered from stratified, securely dated archaeological contexts. The burials from layer 2 at Leang Cappolombo are particularly rare examples of such finds (see Materials and Methods) (*19*). Furthermore, CPL-R2a is the only adult burial of secure provenience recovered from the site, with the other Toalean individuals (*n* = 5) excavated from Leang Cappolombo comprising either young children (∼9 to 14 years old) or infants (see Materials and Methods).

### Maros-LBB-1a

This Late Pleistocene hunter-gatherer is represented only by a right maxilla and molars (M^1^ to M^3^) (Fig. 2, A and B, and fig. S1). The individual’s age at death is estimated to be middle to old adulthood (35+). Although the anterior teeth are missing postmortem, antemortem tooth loss of the right P^3^ is observable. There is pronounced postmortem damage to the alveolar bone of the P^4^, limiting observation of whether the tooth was lost ante- or postmortem; however, resorption of the alveolar margin is identifiable. Periodontal disease of the alveolar bone is present across the three preserved teeth. Notably, Maros-LBB-R1a exhibits severe circumferential wear of the M^1^ to M^3^. A distinct finding is the considerably greater wear of the M^3^ compared to the M^2^, which suggests the repeated storage of a hard spherical object measuring ∼10 to 20 mm in diameter in the back of the mouth, an activity that wore down the molar over time. The M^2^ appears to lack the circumferential tooth wear observed in the M^1^ and the M^3^, suggesting that this individual had a routine habit of moving the spherical object from one part of their mouth to another.

### CPL-R2a

This middle Holocene hunter-gatherer is a mid-to-old adult male represented by cranium, mandible, dentition, and hands (Fig. 2 and fig. S3C). Along with considerably poor oral health (fig. S3D), CPL-R2a presented with essentially the same distinctive tooth wear evident in Maros-LBB-1a. Table S1 shows the wear type of each tooth. Plane wear is present on the anterior teeth and maxillary right molars, which may be a combination of typical masticatory attrition and/or use of teeth as tools. However, this individual also displays pronounced bilateral circumferential wear of the maxillary and mandibular molars, as well as of the premolars and canines (fig. S4). The wear is severe, resulting in root exposure particularly of the M_2_. Circumferential wear of the left mandibular lateral incisor and canine is reminiscent of historically documented dental pipe facets, rounded notches caused by habitual clay pipe smoking (fig. S5, A to C) (*20*), and strongly suggests that a hard spherical object ∼10 to 20 mm in size had been repeatedly held between the teeth and sucked or gently gnawed (probably both). The presence of the circumferential wear across the anterior and posterior teeth in a bilateral fashion further suggests that, as with Maros-LBB-1a, the object in question was positioned at various times throughout different parts of the mouth, potentially as a result of the antemortem tooth loss of various teeth and pain associated with the periapical lesions.

### Hypothesized cause of the dental wear

Maros-LBB-1a and CPL-R2a both present with an unusual dental wear pattern that is inconsistent with typical helicoidal attrition from mastication. To our knowledge, these are the only prehistoric human inhabitants from Sulawesi found to exhibit this distinctive dental pathology, which we surmise was caused by repetitively sucking a hard, round object. Although our sample size is limited to two individuals, it is necessary to reiterate that skeletal remains of the preagricultural population are extremely uncommon. What is more, CPL-2a appears to be the most elderly forager of reliable provenience and dating presently known from the entire Toalean population.

Betel nut chewing behaviors are associated with extreme dental attrition, enamel and root fractures, periodontal disease, antemortem tooth loss, and severe accretion of mineralized plaque deposits (calculus) (*21*). The dentitions of Maros-LBB-1a and CPL-R2a display some of these traits; however, we are unaware of any reports of betel quid usage producing such extreme circumferential dental wear. The teeth of both individuals are stained to some degree, especially the tooth roots and cemento-enamel junctions of CPL-R2a, which is in keeping both with taphonomic processes and the age of these foragers at time of death. However, we can discern no evidence for discoloration consistent with betel quid chewing (fig. S5, C and D). Betel quid usage can therefore be ruled out as the causal agent of the dental pathology (and see below).

**Fig. 3.**
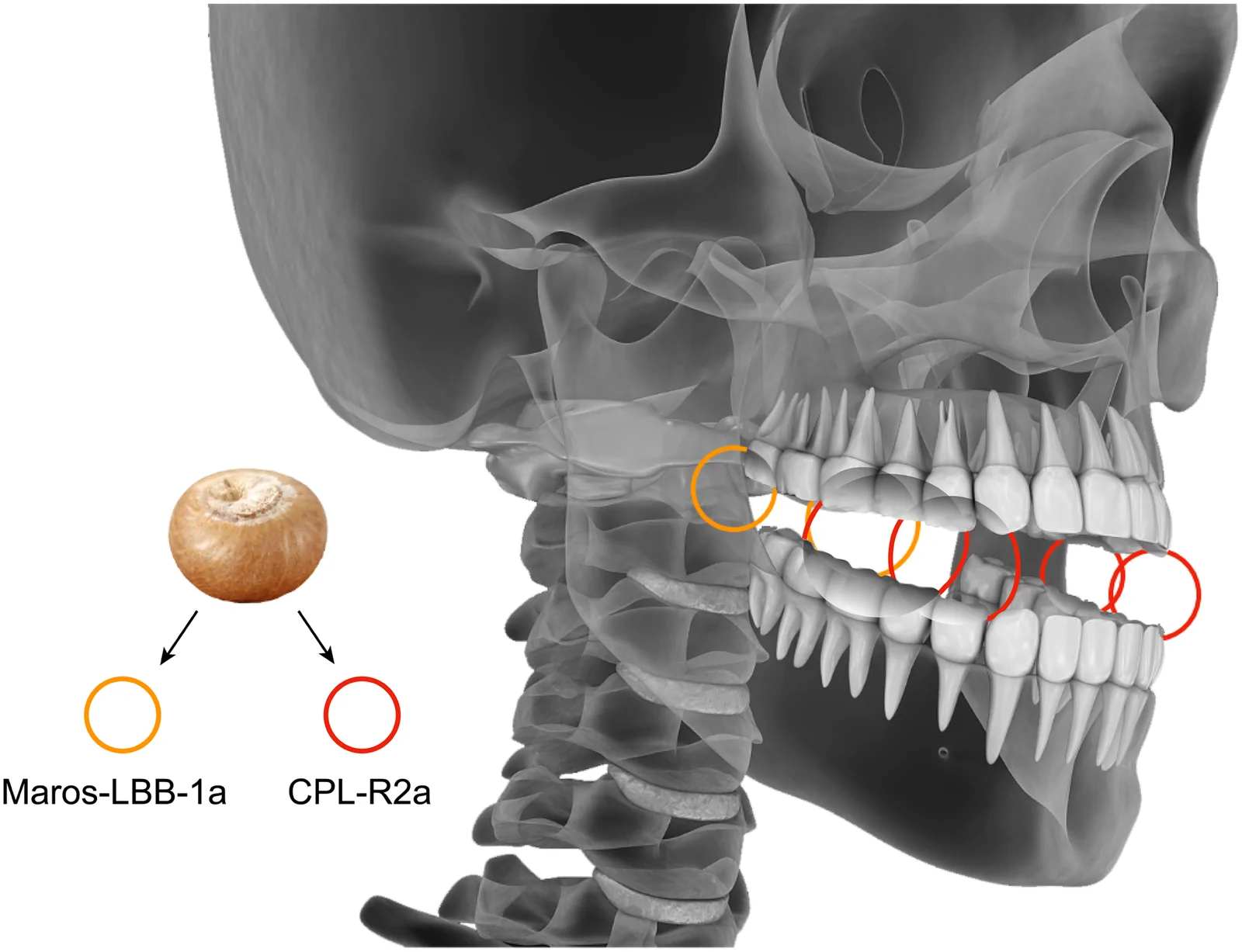
Betel nut sucking positions. The schematic illustration depicts the preferred positions of whole betel nuts held in the mouths of the Late Pleistocene forager (Maros-LBB-1a) and middle Holocene forager (CPL-R2a), as inferred from dental wear analysis. Images licensed from Shutterstock.

The size of the dental wear in Maros-LBB-1a and CPL-R2a is consistent with whole, dehusked areca nut species of smaller size such as *Areca triandra*, “wild *Areca* palm,” which is native to South Sulawesi (*22*). Thus, we surmise that the circumferential tooth wear was a consequence of these individuals’ long-term habit of orally manipulating wild areca seeds, that is, keeping a whole, dehusked betel nut in the oral cavity, clenched between the teeth in various preferred positions (Fig. 3), and continuously moving the tongue and muscles of the mouth around this hard abrasive object and/or gently gnawing it for prolonged periods (we consider both actions to constitute “sucking”). Given that the areca seed has no food value, it is unlikely this inferred practice provided a source of nutrition. Many people develop an ingrained habit of repetitively manipulating nonintoxicating objects in their mouths (e.g., grass stalks); as with the simple oral activity of chewing gum, the rhythmic act of masticating mundane objects may alter brain activity in a beneficial way (e.g., relieving stress, improving cognitive performance) (*23*). However, the oral stimulation practice that we have identified had harmful negative consequences. For such deep wear facets to form in extremely hard dental tissues, Maros-LBB-1a and CPL-R2a must have persisted with this detrimental activity for many years. Such compulsive behavior is suggestive of drug use, if not pathological addiction. Accordingly, we hypothesize that betel nut sucking was motivated by a physical dependence on the areca seed’s chemical compounds. To test our hypothesis, we conducted an experiment to determine whether the biological components of betel nut are released simply by sucking the intact nuts. We also analyzed the ancient teeth for arecoline and other active biomarkers of areca seeds.

## RESULTS

### Muscarinic activity of intact areca nuts

Arecoline is an activator of muscarinic acetylcholine (ACh) receptors and stimulates copious amounts of saliva (*6*). It is also a weak activator of nicotinic ACh receptors found in the brain (*6*). To investigate whether arecoline would be released from sucking intact areca nuts, we tested three different types of these palm seeds (Materials and Methods): (i) dried *A. catechu* nuts, as commonly used in India and Myanmar, and generally known as *supari*; (ii) *A. catechu* nuts from Vietnam that were vacuum packed when fresh and stored frozen; and (iii) *A. triandra*, an uncultivated (i.e., wild) species used in some areas as a substitute for *A. catechu* (*8*). Broths were made by incubating a physiological saline solution overnight with either intact nuts or nuts that had been broken into pieces with a hammer (*supari* and *A. triandra*) or sliced into pieces with a razor (fresh). Broths from intact nuts would be expected to contain only select low–molecular weight components of areca, whereas the broths from the broken nuts would contain all high– and low–molecular weight components that would be in betel quids (*6*). Consistent with this theory, the broths from broken nuts were typically of much deeper color than those from intact nuts (fig. S6).

To evaluate muscarinic cholinergic activity consistent with the release of arecoline, pH-adjusted broths were applied to oocytes expressing human M1 and M3 muscarinic ACh receptors (*24*). The broth from intact *supari* stimulated large transient currents, and receptors recovered sufficiently to be responsive to subsequent application of ACh (fig. S7A, top trace). The application of the broth from broken *supari* evoked smaller peak currents (table S2), but, consistent with previous reports of the sustained activity of high–molecular weight areca molecules (*6*, *24*), there was a large sustained rebound response that prevented any response to the subsequent application of ACh (fig. S7A, bottom trace).

Broth from intact fresh nuts also evoked a large transient response; however, there was also a small rebound and an inhibition of the subsequent ACh-evoked response (fig. S7B, top trace). Notably, the broth from broken fresh areca evoked a very small transient response and although the rebound response was also small, it was sufficient to inhibit the ACh response (fig. S7B, bottom trace). The *A. triandra* broths produced only small transient responses that were similar for both intact and broken nuts (fig. S7C and table S2), whereas the rebound currents and inhibition of ACh were greater for broth from broken nuts.

The control experiments indicated that arecoline was stable in solution for 21.25 hours at 36°C (fig. S7D). In the absence of the other areca components, the receptors retained sensitivity to ACh. Transient peak currents of the individual replicates are presented in fig. S8A and the ACh responses in fig. S8B. The analysis of variance on these data is presented in table S2.

Areca nuts contain high levels of the neurotransmitter γ-aminobutyric acid (GABA), and its presence in the low–molecular weight fraction of whole areca nuts will activate heterologously expressed GABA receptors, whereas components of the high–molecular weight fraction inhibit GABA receptor function (*6*). To determine whether GABA could be released from intact nuts, we applied broths from overnight incubations with intact *supari* or *A. triandra* nuts to cells expressing human GABA type A (GABA_A_) receptors and compared responses to those evoked by control applications of 10 μM GABA (fig. S9). These broths evoked no detectable responses, and control GABA responses were unaffected after the broth applications, indicating that neither GABA nor the high–molecular weight inhibiting components were released from the intact nuts.

To summarize, our experimental data indicate that sucking intact areca nuts would likely release arecoline in quantities sufficient to cause physiological effects (including excessive salivation) associated with betel quid usage. However, while some of the small active molecules from the intact nuts may be released, other small molecules, such as GABA, may not. Intact *supari*-like dried nuts are also relatively unlikely to release pigment and larger molecules that may be associated with toxic effects of areca, although some of these components may be released if fresh nuts are used.

### Biomolecular analysis of the ancient Sulawesi teeth

Two sets of dental samples were analyzed using liquid chromatography–mass spectrometry (LC-MS) (fig. S10; see Materials and Methods). The first set comprised one sample of the fully intact M^2^ still embedded within the maxilla of Maros-LBB-1a (this subsample is denoted Maros-LBB-1a-Gigi) and three small fragments of teeth (Maros-LBB-1a-Gigi1) obtained from this same individual. The second set (CPL-R2a) comprised two fragments of almost a full tooth from CPL-R2a (subsample denoted CPL-R2a-CAPP) and, from the same individual, a fully intact tooth (CPL-R2a-CAPP2) bearing the circumferential dental wear.

Of the first set of teeth with dental wear analyzed, Maros-LBB-1a-Gigi tested positive for arecoline with LC-MS, confirming this Late Pleistocene individual had consumed betel nut (table S3). Maros-LBB-1a-Gigi and Maros-LBB-1a-Gigi1 also had the compounds vanillin and nicotine, which are present in the *A. catechu* seed but also in other plant sources (table S3). Additionally, both samples had eugenol, eucalyptol, cumene, and citral, whereas Maros-LBB-1a-Gigi1 yielded coumarin, farnesene, phytol, stigmasterol, and hexadecanamide. These compounds are all present in *P. betle* leaf, which, as already noted, is a common constituent in prepared betel quids (Fig. 1D). Chewing areca nut with the leaf of *P. betle* has no physiological effect (*25*). It therefore seems likely that these aromatic leaves, rich in essential oils, were consumed separately by early Sulawesi foragers as flavorants and/or for their well-documented medicinal properties (*26*).

The second set of samples (CPL-R2a-CAPP and CPL-R2a-CAPP2) both produced compounds that can be found in the *A. catechu* seed, although CPL-R2a-CAPP2 was the only one that had arecoline present (table S3). Based on the latter result, it is evident that this middle Holocene forager had also ingested betel nut. In the CPL-R2a-CAPP sample, eugenol, eucalyptol, anethole, citral, and cumene were identified, all of which are present in *P. betle*. However, the CPL-R2a-CAPP2 sample did not have eugenol, eucalyptol, and cumene, only farnesene and some of the fatty acids (table S3). GABA was also detected in one of these samples (CPL-R2a-CAPP2) from this individual.

Some notable betel-specific compounds were undetected in this analysis, including guvacine, guvacoline, and arecaidine (table S3). Guvacine and arecaidine are naturally found in very small quantities in betel nut; however, in the presence of slaked lime, large quantities of these compounds form in the betel quid owing to the process of alkaline hydrolysis (*25*, *27*, *28*). It can therefore be expected that, if the two Sulawesi foragers ingested betel nut without slaked lime, there would be arecoline in their dentitions but only very low to undetectable quantities of guvacine and arecaidine. Hence, combined with the absence of clear betel stains on the teeth, our findings are consistent with the hypothesis that the circumferential dental wear was caused by these individuals routinely sucking whole areca nuts.

## DISCUSSION

Our combined biochemical and archaeological findings suggest that extreme dental wear found in two pre-Neolithic foragers from Sulawesi was a consequence of these individuals habitually sucking areca seeds (betel nuts), a nondietary activity that we experimentally demonstrate releases chemical compounds into the brain. While our sample is limited to two individuals (Maros-LBB-1a and CPL-R2a), as previously indicated, these are rare examples of well dated and systematically excavated skeletal remains of preagricultural Sulawesi foragers. Furthermore, both individuals were of relatively advanced age at time of death (CPL-R2a is the most elderly Toalean individual presently known) and thus are more likely to display evidence for dental pathologies and morphological alterations associated with long-term tooth use habits.

The psychoactive properties of areca seeds are likely to have motivated the development of the previously unrecognized custom of betel nut sucking, but it is also possible that this behavior was medicinal in nature [as is betel chewing in some societies; (*7*)]. For example, the increased amount of amylase production stimulated by excessive salivation, an outcome of the release of arecoline into the blood-brain system, would have helped to quickly break down starchy foods (e.g., the starch-rich pith from wild sago palms), thus aiding digestion. It is also likely to have had a cariostatic effect, reducing dental decay. With regard to the latter, both Maros-LBB-1a and CPL-R2a had markedly poor oral health, but the surviving teeth are notably free of caries. On the other hand, the periodontal diseases that these foragers suffered from are far more deleterious than tooth cavities. In the case of CPL-R2a, this individual sucked areca seeds until their teeth had become so severely worn by this activity that the pulp cavities were exposed in several places, resulting in long-term chronic infection. Such a debilitating condition no doubt required ongoing pain management and would have had a negative impact on the physical and mental well-being of this person. It is thus likely that the oral health deficits of this habit exceeded the benefits, unless there was an accompanying effect that reduced the pain and discomfort associated with periodontitis. Concerning the latter, arecoline has demonstrated analgesic properties, and, hence, we suspect that the foragers were intentionally using areca seeds as “painkillers” (*7*, *29*). Therapeutic uses of plant resources may have been quite common among Late Pleistocene foragers in Southeast Asia, owing to the rich plant biodiversity and endemic flora in this region and the particular need for medicinal drugs (e.g., antimicrobial treatments) in the humid tropics (*30*). In any case, it seems possible that Maros-LBB-1a and CPL-R2a became caught in a cycle whereby sucking areca seeds to relieve toothache turned into an increasingly strong habit or pathological addiction as the resultant damage to their dentition became progressively worse.

Our findings allow us to infer the prehistoric roots of one of the world’s most prevalent addictive stimulants. It now seems probable that betel chewing as we know it evolved from a much simpler, older practice of just sucking on the intact nut. While the psychoactive “high” of sucking betel nuts may have been milder than modern quid chewing, the physical dependence was significant enough to cause severe dental damage. It thus seems probable that managing the effects of addiction and other adverse health consequences of drug use was a part of human experience long before the oldest of our most common psychoactive substances, such as alcohol, had emerged.

This research marks a shift in our understanding of human drug history by pushing the timeline back to the Late Pleistocene. It was previously argued that the earliest use of psychoactive drugs dates to the Neolithic advent of agriculture around 12.5 ka (*1*–*3*). Our study demonstrates that betel nut sucking was in use among foraging groups in Sulawesi by 25 to 16 ka, long before the onset of farming. When and where people first began chewing betel nuts in prepared quids, thus giving rise to the modern drug use custom, remains unknown. One possibility, however, is that Neolithic farmers introduced the use of slaked lime to Sulawesi [e.g., as pottery temper; (*8*, *26*)], and, thus, that betel quid usage as we know it originated after the coming of the Austronesians (∼4 ka) and owing to their cultural interactions with the island’s original inhabitants.

## MATERIALS AND METHODS

### Archaeological context and age of the skeletal remains

#### Leang Bulu Bettue

Leang Bulu Bettue (4°59′31.18″S, 119°40′5.53″E) is a valley-floor entrance cave and rock-shelter complex situated in the limestone tower karst lowlands of Maros-Pangkep, South Sulawesi, Indonesia (*15*, *16*). Located at an elevation of 18 m above sea level, the site comprises a rock-shelter with a roof height of 15.6 m and an adjoining chamber that is 27.3 m long, 12.6 m wide, and up to 9.2 m high (*15*, *16*).

Prior studies at Leang Bulu Bettue described human occupational evidence gleaned from excavations in the uppermost portion (∼0 to 2 m) of the deposits (*15*, *16*). That work focused on an aceramic cultural phase denoted phase II (*15*), superseding the cultural phase designations for the site proposed previously (*31*). Phase II is estimated to date to between ∼50 to 40 ka and 16 ka (*15*, *16*, *31*–*34*). The richest cultural deposits within this phase accumulated around the time of the Last Glacial Maximum (LGM) (*15*, *16*). These strata have yielded a chert-based lithic technology (*34*), a partial *Homo sapiens* maxilla (designated Maros-LBB-1a) (*15*), and an assemblage of symbolic material culture items that includes examples of portable art (*32*, *33*) and personal adornments (*31*). Extensive evidence for the processing and use of mineral colorants was also uncovered in the LGM deposit and in deeper stratigraphic contexts dating back to at least 40 ka (*31*).

Dating of phase II was based on ^14^C-dating of plant carbon and freshwater gastropod shells, luminescence-based optical dating methods (feldspar IRSL), and Uranium-series analysis of in situ stalagmites and faunal remains (*15*, *16*, *31*–*34*). The uppermost cultural horizon within the phase II sequence, layer 4a, which yielded the human maxilla (Maros-LBB-1a), is well constrained to between ∼25 and 16 ka by a series of dates obtained from Uranium-series analysis of stalagmites that had formed within these strata (*15*, *16*, *31*).

#### Leang Cappalombo 1

Leang Cappalombo 1 (5°04′29.26″S, 119°57′46.5″E) is situated in the limestone karst uplands of the Bontocani region (Indonesian administrative designation: Pattuku village, Bontocani District, Bone Regency, South Sulawesi). Located 628 m above sea level, the site consists of a cliff-foot niche with a northwest-facing aspect and is 10 m wide and 40 m long. The site itself was discovered in 2015 during an archaeological field survey led by B.H. (*17*, *35*).

Excavations in 2017 (squares TP1, TP2, and TP3) and 2018 (squares U1T1, U1T2, S1T1, and S1T2) reached bedrock at a maximum depth of 90 cm. A sequence of two layers was revealed. The top layer (layer 1) is a very fine, loose sandy sediment (7.5YR 6/3 light brown) mixed with dry vegetal matter. This layer was 20 to 40 cm thick, but, on the north wall of square U1T1, it penetrated to a depth of 70 cm. Several pockets of dry leaf concentrations, 10 to 20 cm thick, were also observed on the north wall of square U1T2 and the east wall of square U1T2. Pottery was found in the deposit, but only in spits 1 to 4 of layer 1. The second layer (layer 2), the basal cultural horizon, is a very fine sand (30 to 50 cm thick) with a texture and color (7.5YR 6/3 light brown) similar to layer 1, but lacking intrusive vegetal materials.

Artifactual remains recovered from layer 2 were confined to materials typically attributed to the Toalean technocomplex of middle to late Holocene South Sulawesi (*18*, *36*). Lithic artifacts were particularly abundant, with a total weight of 30 kg in 2018 and 24.46 kg in 2017. The most dominant artifacts were debitage, totaling 15,044 in number, followed by 95 cores. Retouched flakes included characteristic pressure-flaked Toalean projectiles (Maros points), including the classic variant of this type with serrated margins and a hollow base (*17*, *36*), with a total of 206 artifacts, along with 40 retouched scrapers, and 44 backed artifacts (microliths). Backed artifacts were predominantly found in the upper part of layer 1 (spits 3 and 4), whereas, in the lower part of layer 2 (spits 5 to 7), they were very limited. Conversely, Maros points were more concentrated in the lower part of layer 2 (between spits 5 and 7), which is associated with six human burials.

Concerning the latter, the 2017 excavations in layer 2 uncovered two sets of human remains (denoted CPL-R1 and CPL-R2a), with CPL-R2a found in square S1T1 at a depth of ∼80 cm. Further excavation in 2018 unearthed skeletal elements attributed to another four individuals in layer 2 (CPL-RI3, CPL-RI4, CPL-RI5, and CPL-RI6). These Toalean-associated human remains appear to have been painted with ochreous pigment or sprinkled with powdered ochre (*37*). Aside from CPL-R2a, a middle-to-old adult, the individuals buried at the site comprise young children (*n* = 3) and infants (*n* = 2).

Two samples collected from spit 6 in layer 2 were submitted for radiocarbon dating at the University of Waikato, New Zealand. A bivalve shell sample (square S1T2; 70 to 80 cm depth) yielded a radiocarbon age of 6410 to 6300 cal B.P. (laboratory code: WK-48654), whereas a charcoal sample collected immediately beneath CPL-R2a at a depth of 80 cm, returned an age of 7580 to 7470 cal B.P. (laboratory code: WK-53562). The latter sample was obtained from sediments encasing the cranial portion of this individual. WK-48654 was calibrated using the OxCal v4.3.2 model (*38*) and the IntCal13 atmospheric curve (*39*), whereas WK-53562 was calibrated using the Oxcal v4.4.4 and Atmospheric measurement models (*40*, *41*). Based on these radiocarbon age determinations, layer 2 is inferred to date to between ∼7.6 and 6.3 kyr cal B.P.

### Muscarinic activity of intact areca nuts

The best-studied compounds of areca are the alkaloids: arecoline, arecaidine, guvacoline, and guvacine. The molecular mechanism accounting for the addictive properties of areca nut is still imperfectly understood (*6*, *42*). It is the arecoline, however, that has the most overt physiological effect, excessive stimulation of saliva. Although arecoline is probably not addictive, the salivation that it induces is doubtless a cue that users will associate with the drug. In any case, it seems that addictive effects require the entire nut. Extracts of whole areca nuts are complex mixtures of many components of both low and high molecular weight (*6*, *43*). While it is likely that the neuroactive components are in low–molecular weight fractions, neurotoxic agents are present in the high–molecular weight fraction of whole nut extracts.

#### Commercial reagents

Arecoline and GABA were purchased from Sigma-Aldrich Chemical Company (St. Louis MO, USA), and all other compounds were purchased from Fisher Scientific (Waltham MA, USA).

#### Areca nut infusion

Whole ripe dry *A. catechu* nuts (*supari*), sourced from India, were purchased through eBay from World Wide Export, Bloomingdale, IL. Fresh immature *A. catechu* nuts in their husks and kept frozen, sourced from Vietnam, were purchased through eBay from IHA Beverage, Commerce CA. *A. triandra* mature dry nuts in their dry husks were obtained from a supplier in Muenchen, Germany (rarepalmseeds.com). Husks were removed from the young nuts from Vietnam and from the *A. triandra*. One *supari* nut, weighing ∼13 g, was broken into roughly 0.3- to 0.5-cm cubes with a hammer. Four young nuts were cut into similarly sized pieces with a new razor blade. Six *A. triandra* (∼0.9 g each) were broken into pieces of roughly equivalent dimensions with a hammer.

Whole nuts and broken nuts were placed in individual small beakers with 2 ml of Ringer’s solution [115 mM NaCl, 2.5 mM KCl, 1.8 mM CaCl_2_, and 10 mM Hepes (pH 7.2)] per gram of areca nut, covered tightly, and incubated overnight at 36°C and 140 rpm on a shaker for 21.25 hours. Arecoline (10 μM) in Ringer’s solution was also incubated with them as a control. Each resulting solution was filtered to 0.8 μm. pH was found to be 4.5 to 6.0 and was adjusted to 7.2 with NaOH before testing with muscarinic receptors.

The preparations for tests with GABA_A_ receptors were as follows: one whole *supari* nut and eight whole *A. triandra* nuts were placed in small beakers, covered with 2 ml of Ringer’s solution per gram of areca, covered tightly, and incubated (36°C at 140 rpm for 26 hours). GABA (10 μM) in Ringer’s solution was incubated similarly. After incubation, the areca solutions were filtered to 0.8 μm. The pH of these was found to be 6.2 (*supari*) and 6.8 (*A. triandra*), and these were adjusted to 7.2 with NaOH before testing with GABA_A_ receptors.

#### *Heterologous expression of receptors in* Xenopus laevis *oocytes*

Human a1 and g2(short) GABA_A_ receptor clones were obtained from Philip Ahring (University of Sydney, Australia), and the human b2 GABA_A_ receptor clone was obtained from Trevor Smart (University College London). Human muscarinic M1 and M3 clones were obtained from Tony Morielli (University of Vermont). Subsequent to linearization and purification of the plasmid cDNAs, RNAs were prepared using the mMessage mMachine in vitro RNA transcription kit (Ambion, Austin, TX, USA).

Oocytes were surgically removed from mature *X. laevis* frogs (Nasco, Ft. Atkinson, WI, USA). Frogs were maintained in the Animal Care Service facility of the University of Florida, and all procedures were approved by the University of Florida Institutional Animal Care and Use Committee (approval no. 202300000650). In brief, the frog was first anesthetized for 15 to 20 min in 1.5 liters of frog tank water containing 1 g of 3-aminobenzoate methanesulfonate buffered with sodium bicarbonate. The harvested oocytes were treated with collagenase (1.25 mg/ml; Worthington Biochemicals, Freehold, NJ, USA) for 2 hours at room temperature in calcium-free Barth’s solution [88 mM NaCl, 1 mM KCl, 2.38 mM NaHCO_3_, 0.82 mM MgSO_4_, 15 mM Hepes, and tetracycline (12 mg/liter) (pH 7.6)] to remove the ovarian tissue and follicular layers. Using a Nanoject injector (Drummond, Broomall PA, USA), stage V oocytes were injected with 50 nl of water containing 6 ng of each M1 and M3 subunit RNAs for the muscarinic receptors or 6, 6, and 3 ng for a1, b2, and g2(short) subunit RNAs, respectively, for the GABA_A_ receptors. The oocytes were maintained in Barth’s solution containing additionally 0.32 mM Ca(NO_3_)_2_ and 0.41 mM CaCl_2_. Recordings were carried out 9 days (muscarinic) and 6 days (GABA_A_) after injection.

#### Two-electrode voltage clamp electrophysiology

Experiments were conducted using OpusXpress 6000A (Molecular Devices, Union City CA. USA) (*44*). Both the voltage and current electrodes were filled with 3 M KCl. Oocytes were voltage clamped at −60 mV for cells expressing muscarinic receptors and at −80 mV for cells expressing GABA_A_ receptors to more reliably generate inward currents. The oocytes were bath perfused with Ringer’s solution at 2 ml/min. Test solutions were applied from a 96-well plate via disposable tips. Test applications were 12 s in duration at 2 ml/min followed by 181 s washout periods. The responses were measured as peak-current amplitudes.

Data were collected at 50 Hz, filtered at 20 Hz, and analyzed by Clampfit 9.2 (Molecular Devices) and Excel 2003 (Microsoft, Redmond WA, USA). Data for the GABA_A_ receptors were normalized to the averaged response of the two initial controls for each oocyte. Data were expressed as means ± SEM from six to eight oocytes for each experiment and plotted by Kaleidagraph (Synergy Software, Reading PA, USA). Multicell averages were calculated for comparisons of complex responses. Averages of the normalized data were calculated for each of the 10,500 points in each of the 210-s traces (acquired at 50 Hz), as well as the SEs for those averages. In cases where multiple comparisons were made, a Bonferroni correction for multiple comparisons was applied to correct for possible false positives.

### Biomolecular analysis of ancient human teeth

The aim of the molecular and chemical analysis was to compare residues on the ancient human dental remains from Sulawesi with the chemistry of the *A. catechu* and *P. betle* plants (*11*, *45*–*53*). Recovering molecular and chemical evidence of betel nut use in the distant past requires comparing the chemical residues on ancient dental remains with the chemistry of the *A. catechu* and the *P. betle* plants, both of which are relatively well-known. Chemical characterization of archaeological materials, interpreted as betel quid chewing in the past, has been established previously (*10*, *12*, *13*). Most prior studies along these lines have used destructive techniques for the extraction of biomolecules (predominantly alkaloids), either by powdering the whole tooth (*54*, *55*), undertaking dental tissue separation and powdering (*10*, *56*, *57*), or surface removal using a drill (*12*, *58*), or applied their analyses to the dental calculus (*13*, *59*). This study employed an extremely minimally invasive approach (using a soak technique) for extracting biomolecules from ancient dental remains, with no cutting, grinding, or even acid-etching carried out.

In this study, each tooth sample was rinsed with ethanol and then placed in acetonitrile, methanol, or a combination of both for the removal of the compounds found within the residue trapped on the surface and within the tooth. Enamel is very hard and has a relatively smooth surface with some pits and scratches, while dentin is softer with microtubules throughout. The tooth samples were submerged in the solvent, exposing the solvent to the tooth surface and inside the root canal. All samples were soaked for a minimum of 1 week in the solvents. Once the extraction was completed, the samples were placed into sterile autosampler vials with 200-μl glass inserts in preparation for LC-MS. The LC-MS used is a Vanquish Horizon UHPLC (ultrahigh performance liquid chromatography) coupled with an Orbitrap Exploris 120 (quadrupole) mass spectrometer.

The samples were directly applied to LC-MS on the Exploris 120 Orbitrap at Griffith University (Nathan campus), Brisbane. No modern betel nut (*A. catechu*), areca seeds of other species used as betel chewing substitutes (e.g., *A. triandra*), or betel leaf (*P. betle*) have ever been analyzed in this laboratory or on this column. A normal injection mode with 2.5-μl injection volume was used with a static spray voltage and gas mode. The sampler temperature was 50°C and the column temperature was 50°C. The runs were in positive polarity with the Orbitrap resolution of 60,000, a scan range from 85 to 950 mass/charge ratio and 100-ms scan time. The filter type was a dynamic exclusion. The normalized AGC target was set to 200%. The pump pressure had a lower limit of 73 psi and an upper limit of 8700 psi. The draw speed was 3 μl/s. A 12-min run time was used. Two mobile phase solutions were used: a 1% formic acid in water (solution A) and 100% acetonitrile (solution B). The solution-run conditions were set to 0 to 7.5 min using a 5% solution B; 7.5 to 10 min using 95% solution B; and 10 to 12 min back with a 5% solution B. The runs were performed on a Accucore RP-MS C18 reverse-phase column. The column is 100 mm in length with a 2.1-mm internal diameter, with a 2.6-μm particle size. A standard pressure mode was set, and the flow rate used was 0.4 ml/min. This column had recently been purchased and none of the compounds identified in this study have ever been identified through this column previously. The data generated were analyzed using Xcaliber software and compound identification was performed using the Compound Discoverer v3.2 software with mzCloud, ChemSpider, mzVault, and MassList databases. Solvent blanks were included with each run and any compounds identified in the blanks were subtracted from the data analysis of the sample runs.

### Osteological and dental wear analysis

Age-at-death and sex estimation followed standards outlined elsewhere (*60*). The poor preservation of the individuals meant that age at death was restricted to dental wear. Dental wear is a relatively poor indicator for age at death in comparison to more reliable standards for the pubic symphysis and auricular surfaces of the pelvis and generally only provides an estimate of the life stage (young, middle, or old adult), as opposed to a more precise estimate of age at death. Artifactual dental wear was observed in both individuals, which suggests acceleration of typical dental attrition from mastication that dental wear standards for age at death are based on. For this reason, we provide a very broad estimate of life stage of both individuals. Sex estimation could only be conducted on one individual (CPL-R2a) and followed published standards for weighted estimation of secondary cranial characteristics (*61*). Dental pathology and interpretations of artifactual dental wear followed (*62*). Medium to severe calculus is present on the right M^2^, M^1^, P^4^, left P^4^ and M^1^, right M_1_, and left M_2_.

## Supplementary Materials

### This PDF file includes:

Supplementary Text Figs. S1 to S10 Tables S1 to S4 References
