# Supplementary material for: Betel nut sucking is the earliest recorded evidence for the habitual use of a neuroactive plant drug
Source: Sci Adv. 2026 Sep 9;12(37):eaei1901. doi: 10.1126/sciadv.aei1901 (PMC13557070; doi:10.1126/sciadv.aei1901)
Supplement: Supplementary file 1 [file sciadv.aei1901_sm.pdf]

Science Advances
AAAS

Supplementary Materials for

Betel nut sucking is the earliest recorded evidence for the habitual use of a neuroactive plant drug

Adam Brumm *et al.*

Corresponding author: Adam Brumm, a.brumm@griffith.edu.au

Sci. Adv. **12**, eaei1901 (2026)
DOI: 10.1126/sciadv.aei1901

This PDF file includes:

Supplementary Text
Figs. S1 to S10
Tables S1 to S4
References

SUPPLEMENTARY TEXT

A review of betel quid usage in Southeast Asia and Pacific prehistory

We conducted an extensive analysis of prehistoric evidence of betel chewing from human skeletal remains in Southeast Asia-Pacific, along with the evidence for habitual chewing or decoration (i.e., cosmetic tooth-dyeing) available, and ascertained whether secure dating was conducted. While there has been archaeobotanical evidence for *Areca* spp. dated reportedly to 13,000 years ago (*63*) for East Timor by Glover (1977) (*64*), this finding was later clarified to be associated with layers dated to 2500 BP, with the original data being that of the basal layer of occupation at the site of Uai Bobo 2. Zumbroich (*8*) also points out that, in subsequent publications, reference to *Areca* spp was replaced with an ambiguous reference to *Piper betle* in the deepest layers (now dated to 11,400 BP) (*65*).

Another early date for wild phenotype *Areca* spp is reported for Spirit Cave in northern Thailand in a layer radiocarbon-dated to ~8700 BP (uncalibrated) (*66*). The archaeobotanical remains documented include one carbonized fruit petiole (leaf stalk) and two portions of husk of uncarbonized fruit, of which some of the samples appear to be found within a layer that is dated to 7600 BP. It is not clear in Yen's (*67*) archaeobotanical report of Spirit Cave which samples studied belong to which stratigraphic layers. Subsequent reports by the lead excavators that are absent from the original archaeobotanical study cite the presence of seed cases of *Areca* spp and *Piper* spp in basal layers with no explanation as to how these seeds were identified and by whom (*66*).

Archaeobotanical evidence in and of itself does not provide the necessary information required to assess how it has been used. We argue that the minimal botanical remains found in comparison to other plants species at the site, such as edible *Canarium* spp, further negates any confident attribution of the areca plant remains to deliberate and consistent importation by human inhabitants to the site. While oral uses of areca include for medicinal purposes such as an anthelminthic, sedative, stimulation of digestion, anti-diarrhetic prepared through curing, fermenting, baking, drying, and boiling (*7*), these plants have various traditional uses that do not necessarily involve mastication. For example, the husk is used to brush teeth and create board and paper, the palm used for building materials, and the nut used to dye clothes (*7, 68*).

Human dental remains provide more direct evidence for oral consumption or decorative staining of teeth, but without chemical confirmation the evidence for areca nut remains inferential. As Zumbroich (*69*) demonstrates, there are more than 60 plant species used in Southeast Asia for blackening teeth for cosmetic purposes. Therefore, without the identification of chemical signatures of areca-specific tannins via mass spectrometry, evidence of dental staining is merely suggestive of betel nut and/or betel quid use.

A great variety of reported evidence for the practice of betel nut chewing exists throughout the Asia-Pacific region (Table S4). Currently, the most robust evidence of betel nut use in the bioarchaeological record is from late Neolithic Mainland Southeast Asia (MSEA) at approximately 3500–3000 BP (*13*). The dentitions from three individuals from Nong Ratchawat in central Thailand exhibited no evidence for dental staining, but mass spectrometry revealed key tannins including arecoline which are strongly supportive of habitual betel nut chewing. A contemporary date for staining of posterior teeth has been reported by Willis in her PhD thesis for An Son, a late Neolithic site in southern Vietnam (70). However, a recent (2024) analysis of the assemblage by M.V. found that the 50% of staining reported for subadults at An Son

attributed by Willis to exogenous staining from betel nut appears more consistent with postmortem staining of poorly mineralized teeth, as the staining matches the color of the surrounding bone and developmental defects of enamel were common.

Crucially, no secure human skeletal evidence for betel nut use (whether habitual or due to deliberate staining) is dated older than the two major agricultural expansions of MSEA and Island Southeast Asia (ISEA): the expansions of agricultural mainland post-Hoabinhian and Austronesian communities. However, a few papers with less secure dating or indeterminate evidence for betel nut chewing do hint at a pre-Neolithic origin. Samper Carro et al. (*71*) have reported reddish staining on the teeth on the buccal aspect of posterior teeth of an individual from Tron Bon Lei, Alor Island (Indonesia), and which is securely radiocarbon-dated to 7500 BP, but they take a cautious approach and indicate they will assess whether this staining is caused by betel nut chewing via GC-MS in the future. Unfortunately, no images are provided to assess their reports. Evidence from Guar Kepah in Malaysia may date as far back as 5700 BP based on a radiocarbon date from a single shell. However, the burials themselves have yet to be radiocarbon-dated. Substantial erosion of all three middens that make up Guar Kepah, and the presence of pottery throughout the stratigraphic layers, does suggest that the burials assessed by Bulbeck in 2005 (*72*) may be more recent. Ceramics only emerge in ISEA from 4000 BP and are typically associated with the Austronesian expansion (*73*). The skeletons studied by Bulbeck were excavated in the late 19th to early 20th century and therefore no stratigraphic control was implemented in the excavation processes, necessitating the need to securely date the burials from the human remains themselves. These individuals also demonstrate skeletal phenotypic affinities to both Australo-Papuan and Austronesian ancestral and descendant communities (*72*); therefore, a pre-Neolithic date for the betel nut staining remains challenged until AMS dating is conducted on these burials (only returned to Malaysia from the Netherlands in early 2025).

Another frequently cited case of potential pre-Neolithic dental evidence for betel nut use was reported for an individual from Duyong Cave in Palawan, Philippines, by Fox (*74*) more than 50 years ago. The reported evidence from a single burial radiocarbon-dated to 4630 BP is presented purely as evidence of ‘staining’ without other descriptors provided, thus this claim cannot presently be verified. Other potential causes for dental staining including hematite use which is a known practice in pre-Neolithic ISEA as plausible alternative explanations (*75*). Pietrusewsky et al. (*76*) attribute staining on all surfaces of the teeth of individuals from Nankuanli East in Taiwan radiocarbon dated to 4800–4200 BP to betel nut. Published images appear to show red-brown staining, and, as is the case with CPL-R2a, severe circumferential attrition. The site seems to be culturally aligned with Austronesian-speaking people. Radiocarbon dating of ceramics are not associated with the burials, which does mean the dates are not securely tied to the betel nut cases (*77*).

More frequent and secure reports of betel nut staining from ISEA appear to emerge approximately around 3000 BP following the expansion of Austronesian-speaking voyagers. For example, various sites in East Nusa Tenggara dated around 3000 BP support a widespread cultural practice of betel nut use at least in this region of the Lesser Sunda Islands (*78*).

For MSEA, betel nut staining appears in multiple archaeological sites from around 2500 BP and the rise of polities during the Iron Ages. Oxenham (*79*) identified that 40% of individuals from a composite Metal Period sample from northern Vietnam show signs of deliberate staining on the labial surfaces of anterior teeth. While Oxenham (*79*) does not stipulate whether any betel nut cases predate 2500 BP, and at which sites in the composite sample betel nut staining was

identified, he does report that more than 80% of the sample post-dates 2500 BP. For both MSEA and ISEA, as expected, evidence of habitual chewing predates that of deliberate staining for aesthetic or identity purposes.

The radiation of dates outward from the Philippines, Malaysia, and Indonesia towards MSEA and Oceania does suggest that the island archipelagos and peninsular Southeast Asia served as the initial cradle for the emergence of betel nut chewing cultures, before diffusing alongside trading, migration, and voyaging possibly with Austronesian cultures. However, the addition of the two Sulawesi cases to this research identifies a non-Austronesian origin within Wallacea for a hitherto unrecognized early stage in the development of this widespread drug use habit. Therefore, rather than areca nut-using practices characterizing Austronesian expansion from Taiwan, deep historical evidence for aspects of habitual betel nut use instead supports Indigenous innovation that was exchanged during contact with migrant Austronesian-speaking people and assimilated into Austronesian-related cultures. That evidence of deliberate staining is only present in MSEA following rich trade, exchange and migration of Austronesians on the borders of present-day central Vietnam from around 2500 BP (known as the Sa Hyunh culture), does lend itself to the plausibility that deliberate dental staining practices have roots within Austronesian-speaking communities.

Three loose teeth argued by Kirch (*80*) to possibly be associated with betel nut from the Lapita period contribute potentially the earliest evidence for betel nut use in Oceania. However, the burials were highly fragmented, and radiocarbon dates extend as recent as 1500 BP. The earliest secure dates for potential betel nut use in Oceania are reported by Fitzpatrick et al. (2003) (*81*) for Chelechol ra Orrak, Palau, Micronesia, suggesting use of areca nut for both deliberate staining and habitual use from at least 2300 BP. While the authors indicate the likelihood that the staining was caused by habitual use, the richness of the color on anterior teeth, with minimal tooth wear does suggest deliberate staining practices may have also been present. Earlier dates for potential betel nut staining from Ucheliungs Cave in Palau are claimed to be approximately 3000 years old, but these dates appear unsecure as many of the teeth are apparently surface finds collected by tourists (*82*). Calibrated radiocarbon dates from human bone also appear to be around 2340–1300 BP (*82*). Dates from sites across Oceania are generally consistent with an emergence of betel nut use at around 1500–1000 BP, coinciding with the rise of the so-called Latte Period in Micronesia, and later post-Lapita settlements in Near Oceania (*83–86*).

What has been highlighted by both the research we report here and by Moonkham et al. (*13*) is that the focus on more cost effective macroscopic identification of betel nut staining—which has allowed recognition of the ubiquitous use of areca nut throughout prehistory in Southeast Asia and Oceania—is likely leaving vast gaps in recognition of the behavior in Southeast Asia's ancient past, especially of habitual use as staining does not necessarily occur. Nong Ratchawat serves as a cosmopolitan nexus for trade, exchange, and craftmanship (*13*), yet no other signs of betel nut use have been identified in other major Neolithic and early Bronze Age sites of Thailand and Vietnam such as Ban Non Wat (*87–89*), Khok Phanom Di (*90*), and Man Bac (*91*). Of course, these claims remain to be tested using mass spectrometry. Severe and accelerated attrition of teeth at Khok Phanom Di (*90*) (4000–3500 BP) does suggest the necessity for further research on these foundational archaeological sites in MSEA using mass spectrometry techniques.

Provenance

The human skeletal remains and associated archaeological assemblages from Leang Bulu Bettue were recovered in 2017 during joint BRIN-Griffith University excavations. The skeletal element in question (Maros-LBB-1a) was identified by the team's in-field specialists and later assessed and analyzed by Y. Kaifu, D. Bulbeck and M.V. and P.Y.. Dating work involved multiple approaches (radiocarbon, luminescence, and Uranium-series analysis). The human skeletal remains (CPL-R2a) and associated archaeological assemblages from Leang Cappolombo 1 were recovered in 2017 during Indonesian-led (University of Hasanuddin [UNHAS]) excavations. The skeletal elements were analyzed by Fakhri and M.V. and P.Y.. Dating was focused on the radiocarbon method. Skeletal elements and assemblages from both sites will be housed at the UNHAS campus in Makassar, and can be accessed for analysis with relevant permissions.

Figures

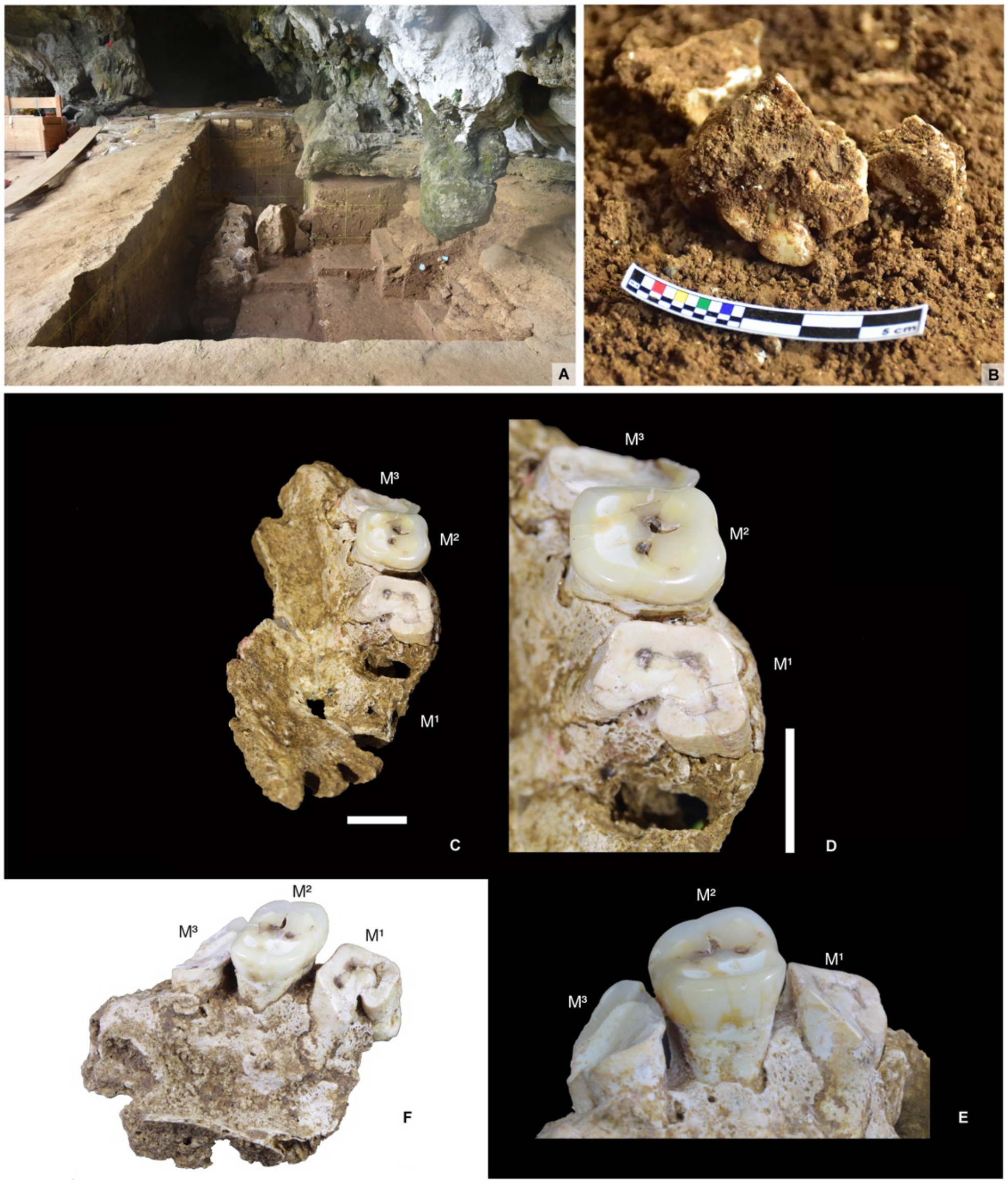

Fig. S1: The Late Pleistocene individual (Maros-LBB-1a) from Leang Bulu Bettue. (A) Excavated trench in the rock-shelter at Leang Bulu Bettue; (B) the human maxilla fragment (Maros-LBB-1a) *in situ*; (C-D) detail of the right maxilla (after reconstruction) and the extent of dental wear on the M^1 and M^3; (E-F) detail of the lingual sides of the extant first to third right upper molars (M^1 to M^3), showing the extreme degree of occlusal attrition on the M^1 and M^3; the image in (F) was taken prior to reconstruction. The teeth are all relatively unstained (the reddish-pink substance visible in some images is residual casting silicon), the M^2 presenting with only mild dental calculus. The scale in (C-D) is 10 mm. Photo credits: R. Sardi (A-D).

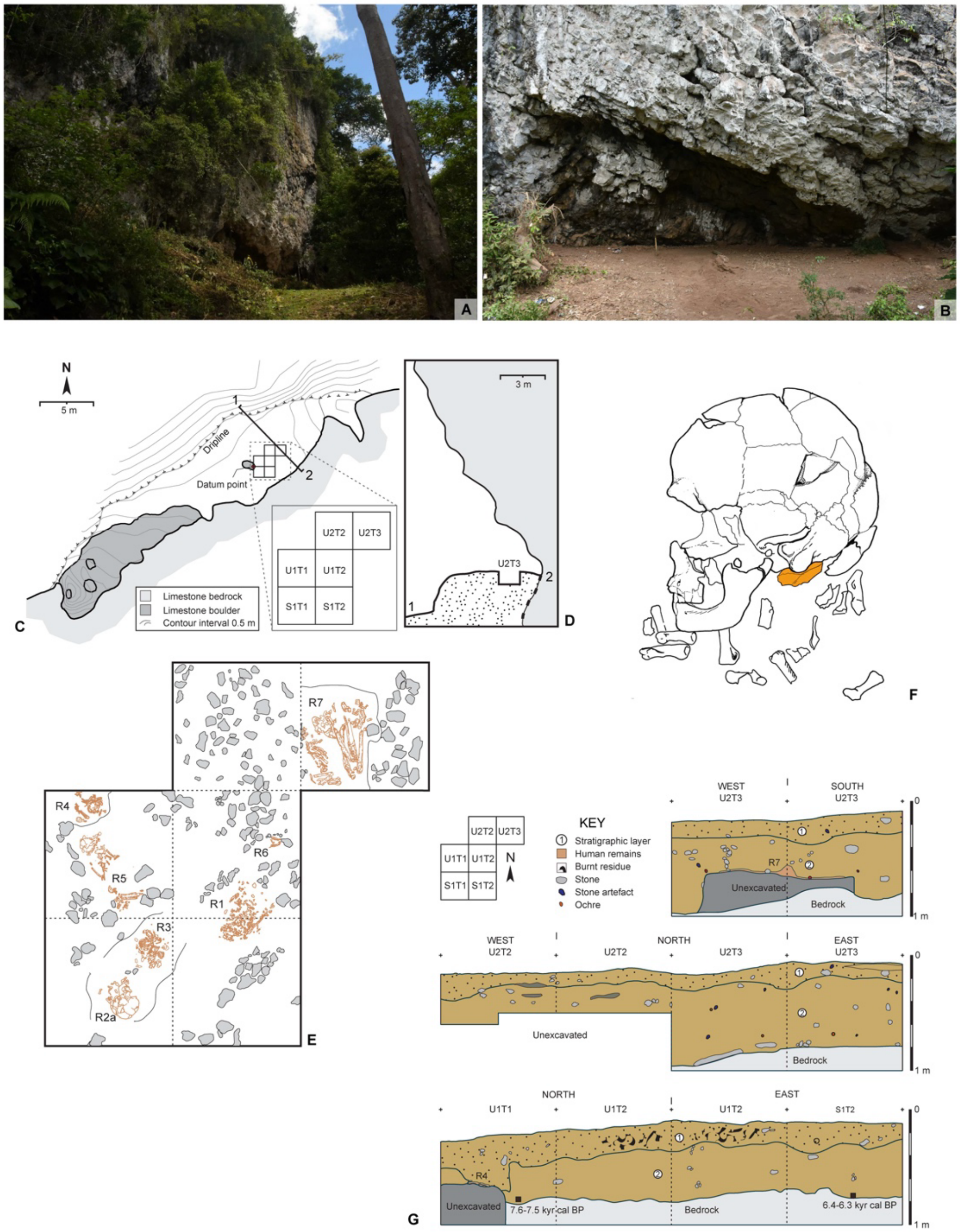

Fig. S2: Archaeological context of the Middle Holocene individual CPL-R2a. (A-B) Leang Cappolombo 1 rock-shelter; (C) site plan showing the location of excavation squares; (D) cross-section of ground floor surface and overhanging cliff-face (1-2 in (C-D)); (E) distribution of skeletal remains of individuals (CPL-R1 to CPL-R7); (F) plan drawing of the *in situ* cranium and post-cranial elements of individual CPL-R2a and an associated flaked stone artefact (shaded orange); (G) stratigraphic profile and dates (kyr cal BP = thousands of calibrated radiocarbon years before present). The skeletal remains (CPL-R1 to CPL-R7) were all recovered from the Toalean-associated layer 2.

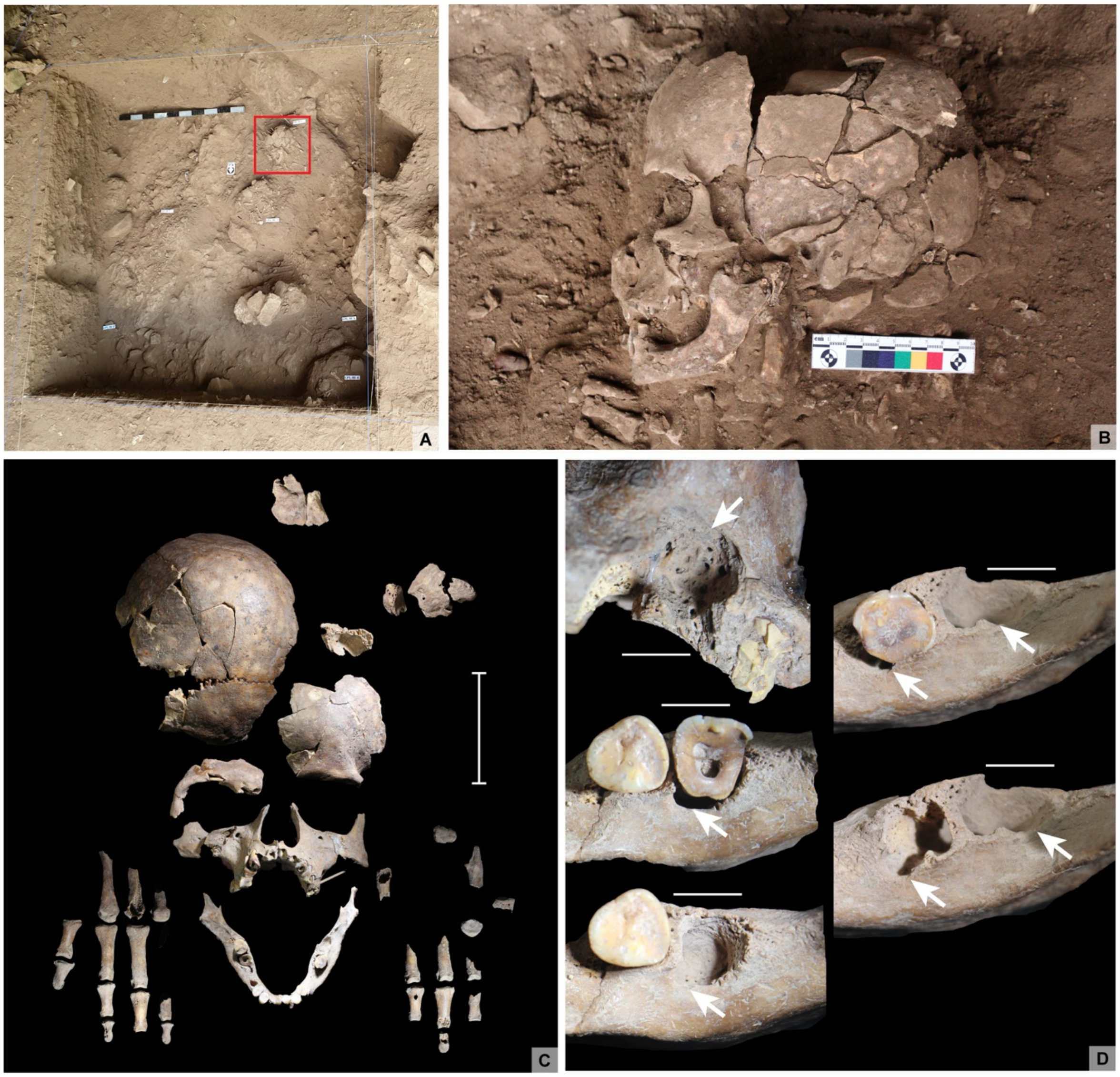

Fig. S3: Middle Holocene individual (CPL-R2a) from Leang Cappolombo 1. (A) Archaeological trench showing the *in situ* burial of CPL-R2a (highlighted by rectangle); (B) detail of *in situ* cranium of CPL-R2a; (C) skeletal preservation of CPL-R2a; (D) periapical lesions and antemortem tooth loss of CPL-R2a. Marked periodontal disease of the alveolar bone is present on both the maxillae and mandible. The individual presents with antemortem tooth loss of the right I^1, I^2, and C^1, left I^2, left M^1, right I_1, I_2, P_4, M_1, and left P_4, and M_1. Three periapical lesions are present at the roots of the left M^1 (11.06 x 11.07 mm), right M_2 protruding into the root space of the M_3 (8.5 x 8.5 mm), and left M_2 protruding into the root space of the M_3 (12.7 x 8.6 mm) (white arrows). All three periapical lesions present with partially remodeled margins, indicating long-term chronic infection. Remodeled new bone and porosity in the left maxillary sinus is consistent with infection of the periapical lesion of the left M^1 infiltrating the sinus. Scale bar in (C) is 80 mm; scale bars in (D) are 10 mm.

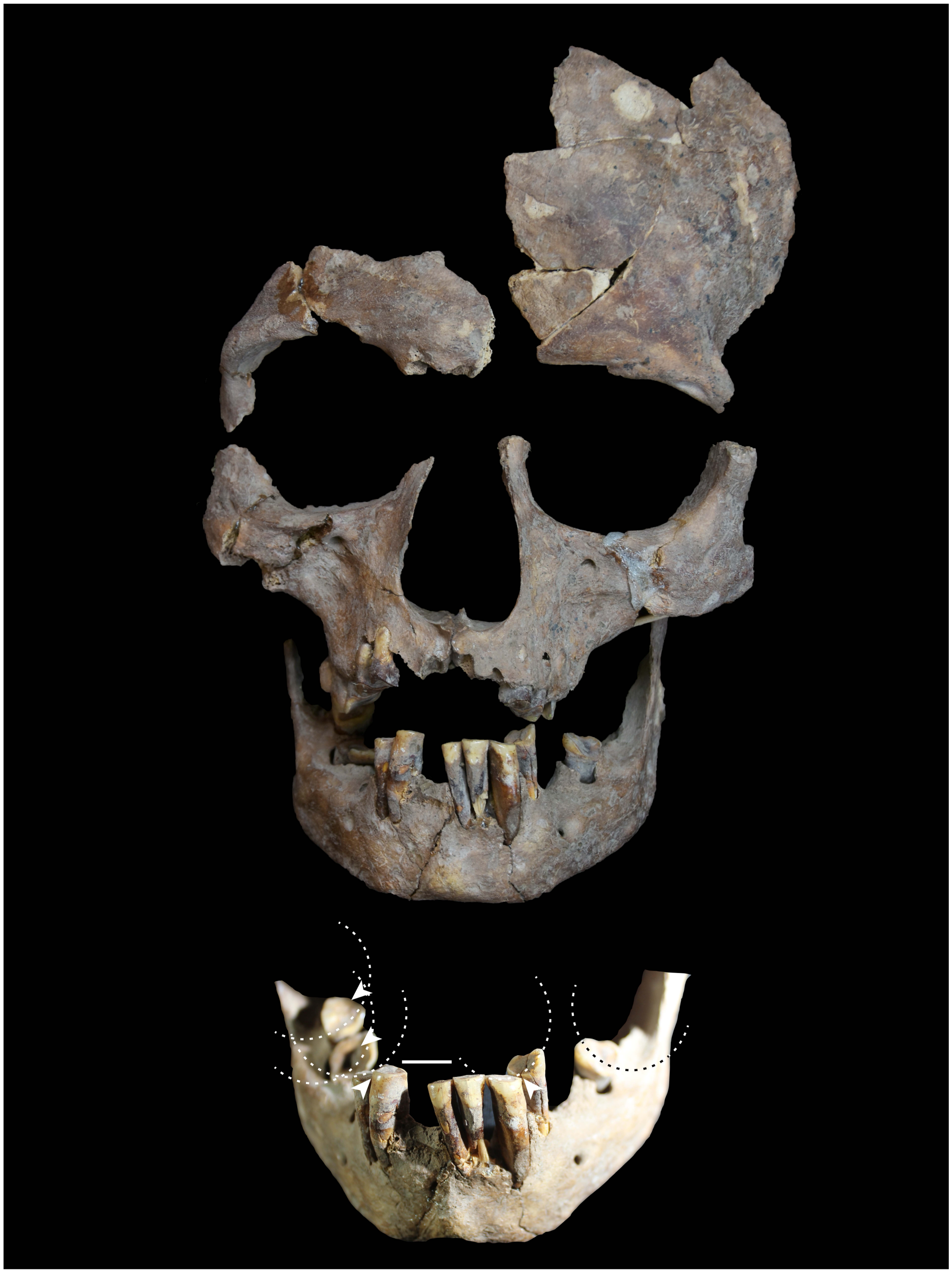

Fig. S4: Cranial reconstruction of CPL-R2a presenting the relationship between maxillary and mandibular tooth wear. Circumferential wear of CPL-R2a's mandibular dentition has been delineated. The tooth wear is highly consistent with a spherical object, with at minimum five positions in the mouth where the object was habitually held and sucked/gnawed on. Antemortem tooth loss of the left and right M_1 molars suggest these may have been former regions where the object was held, since lost due to severe attrition.

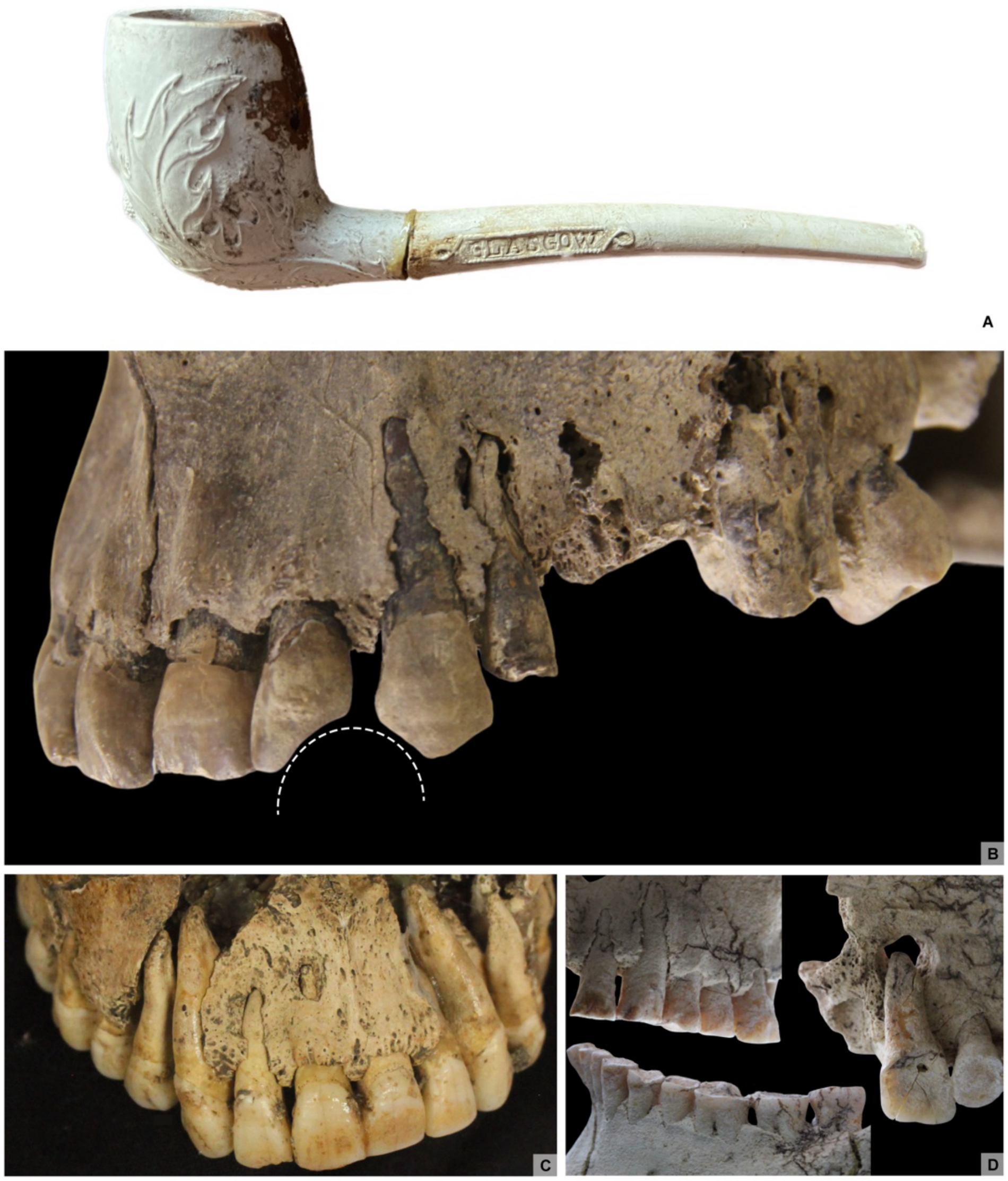

Fig. S5: Clay pipe facet and betel stains in human teeth. (A) Historical period clay pipe; (B) clay pipe facet (indicated by dotted half circle) formed in the dentition of an adult male (St. John's Cemetery, Milton, New Zealand ~1860–1920 CE). Such facets formed in the dentition of long-term tobacco smokers through habitual sucking/gnawing of the abrasive stems of clay pipes; (C-D) dental stains from chewing betel nut (adult males from Nagsabaran (D) and (E) Napa in the Philippines ~2 ka). Dental stains appear light reddish-brown in color and impact lingual and labial surfaces, as well as anterior and posterior teeth indicative of habitual betel chewing rather than deliberate staining. The Napa individual also displays marked circumferential tooth wear. Images by M.C.L. (A) and H. Buckley and M.V. (B-D).

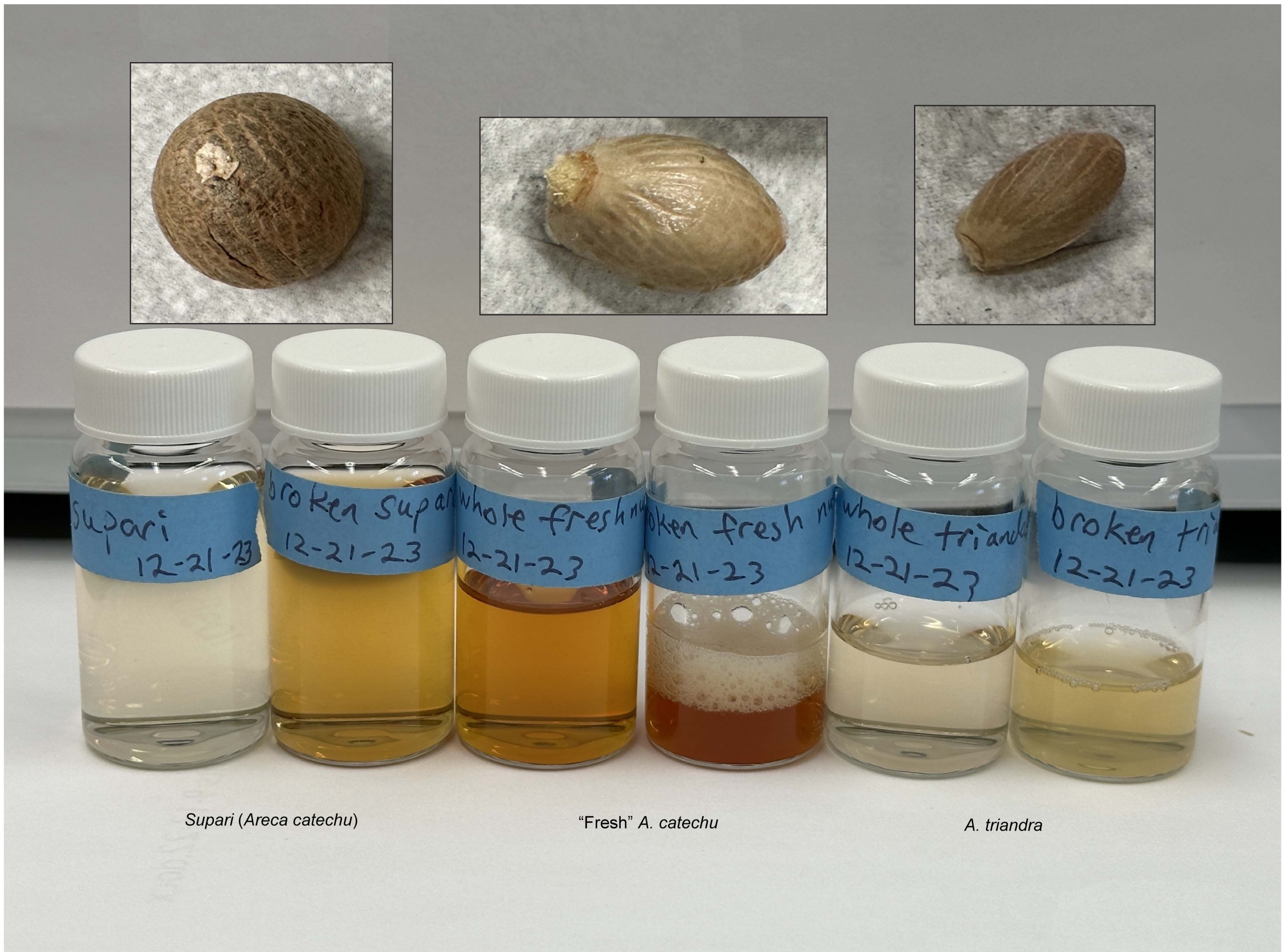

Fig. S6: Areca nuts and solutions used after filtration; dried *Areca catechu* (*supari*), recently thawed, 'fresh' *A. catechu* nuts, and fresh *Areca triandra*. Nuts are shown above, and below are broths prepared by overnight incubation with either the intact nuts or broken (chopped) nuts.

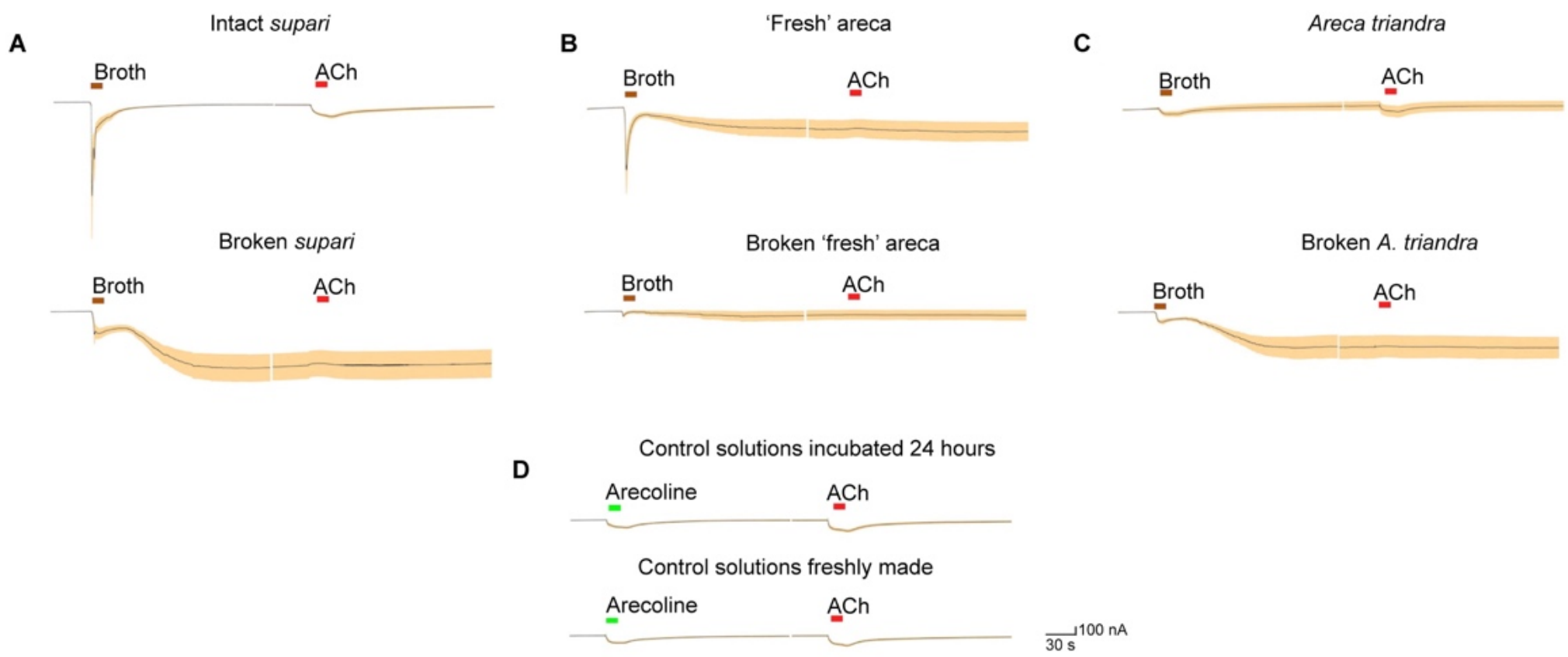

Fig. S7: Averaged raw data traces obtained when broths shown in Fig. S5 were applied to cells co-expressing M1 and M3 muscarinic receptors. (A) *Supari* preparation; cell numbers were seven for intact *supari*, seven for broken *supari*; (B) recently thawed 'fresh' *A. catechu* nuts; cell numbers were six for intact fresh nuts, seven for broken fresh nuts; (C) *Areca triandra* preparation; cell numbers were seven for intact *A. triandra*, and seven for broken *A. triandra*; (D) control data with applications of 10 µM arecoline and 10 µM muscarinic acetylcholine (ACh) control. The arecoline solution for the upper traces was incubated for 21.25 hours at 36˚C to confirm its stability in solution.

A

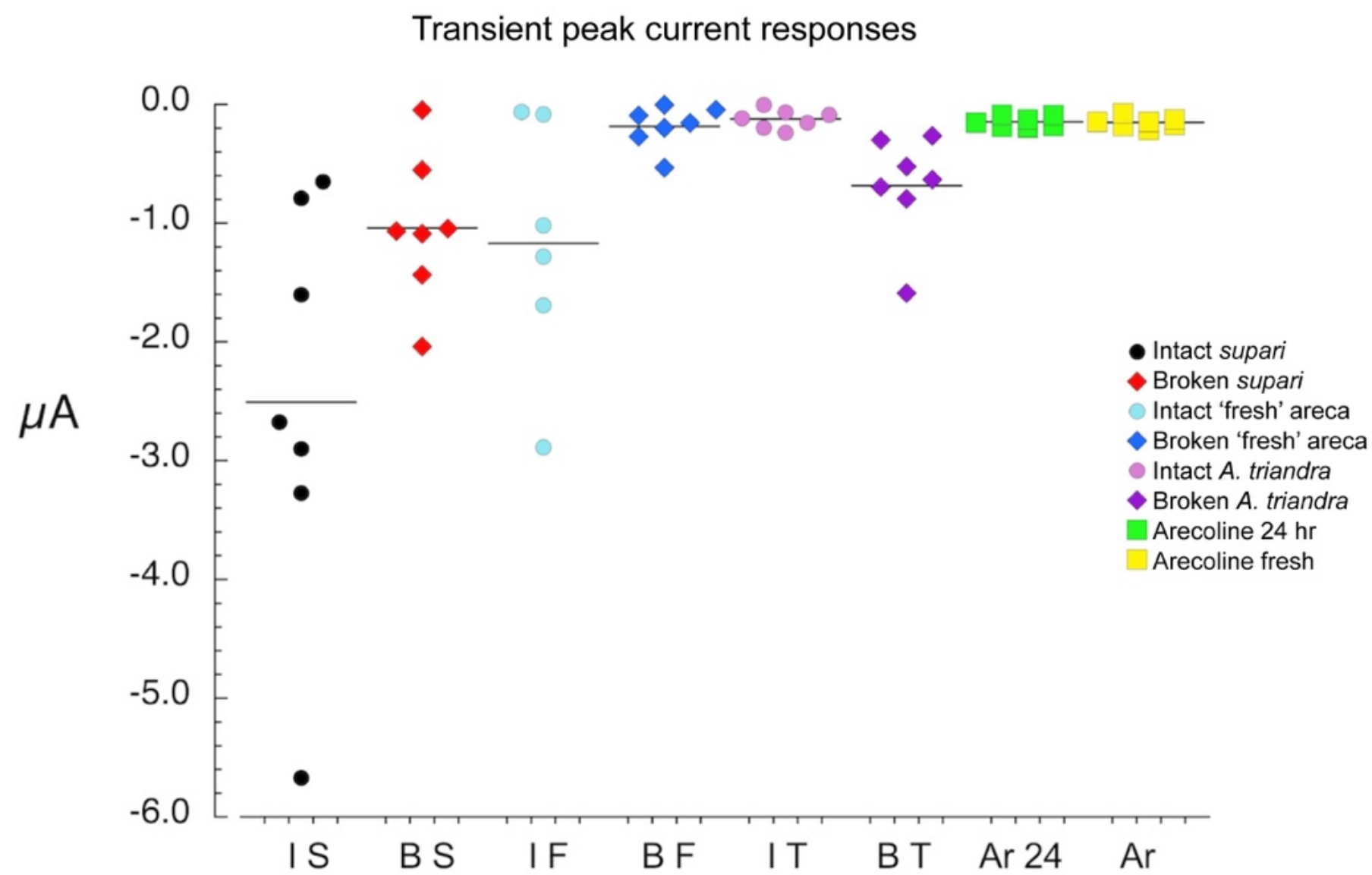

B

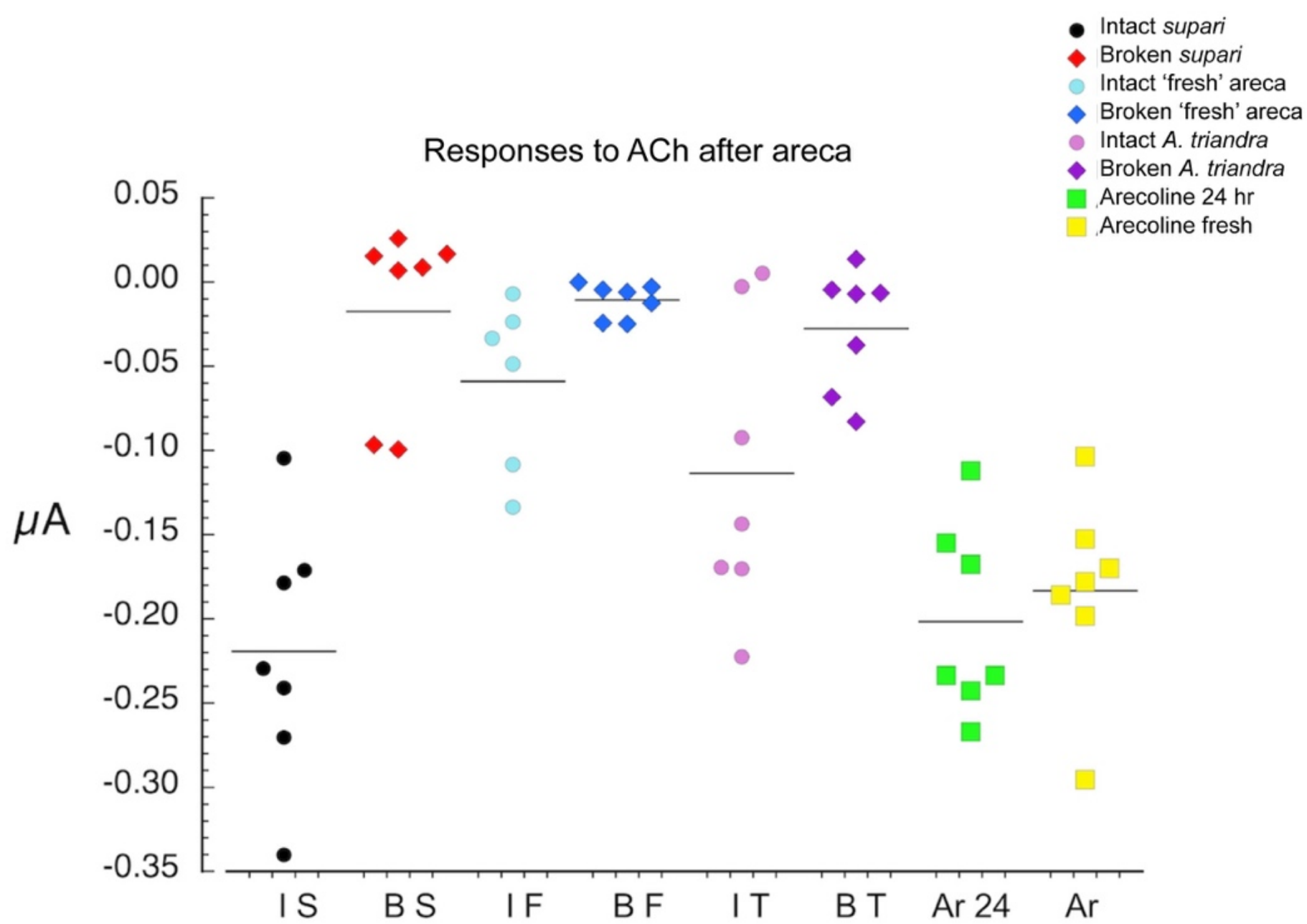

Fig. S8: Transient peak currents of the individual replicates and the muscarinic acetylcholine (ACh) responses. See also Table S2.

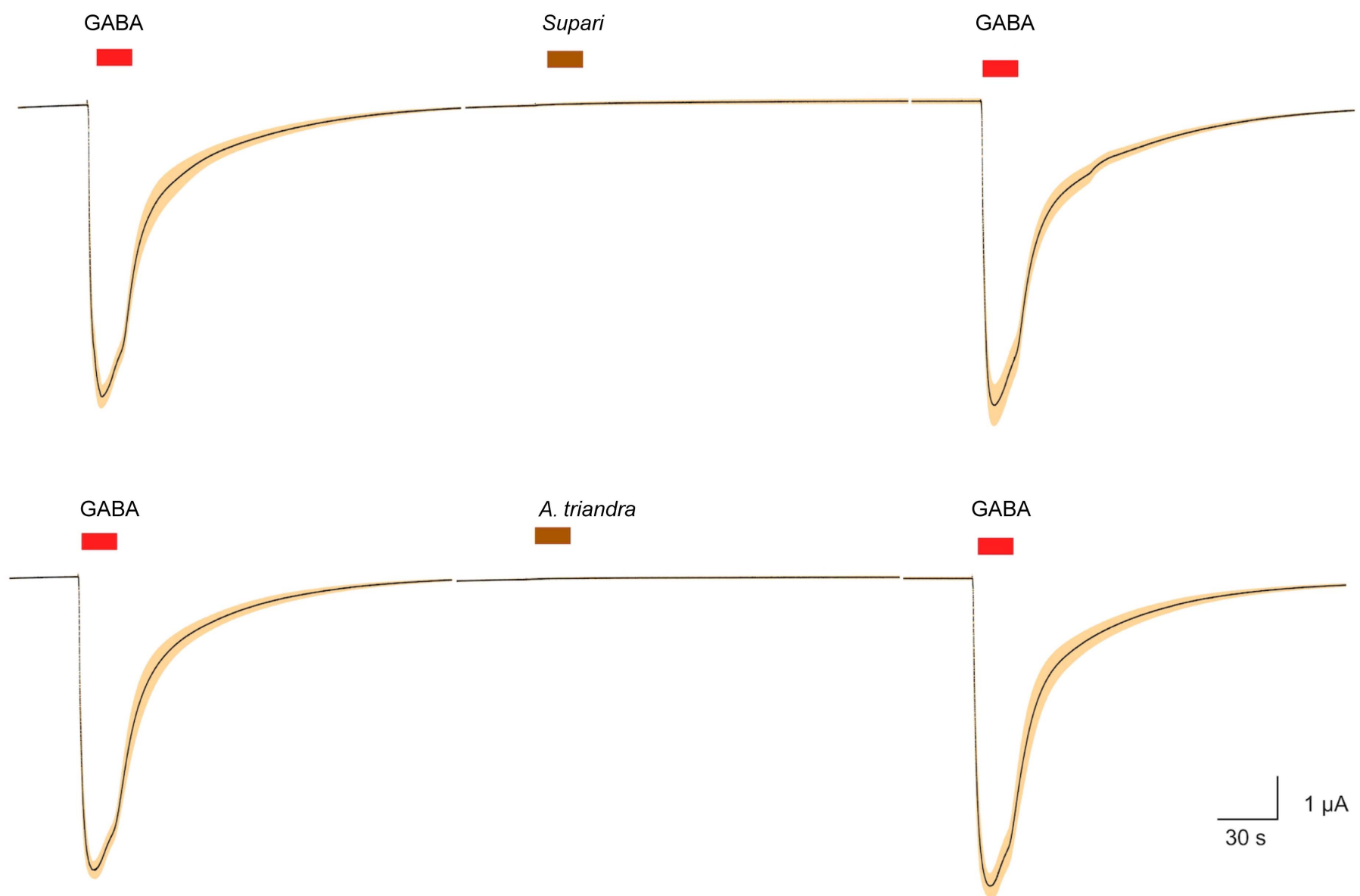

Fig. S9: Averaged responses of cells (n = 8 in both experiments) expressing $GABA_A$ receptors to control applications of 10 μM gamma-aminobutyric acid (GABA) or broths made from incubations with intact *supari* (*Areca catechu*) or *Areca triandra* nuts. Cells were voltage-clamped at a potential of -80 mV so that the outward flow of chloride ions appears as inward currents.

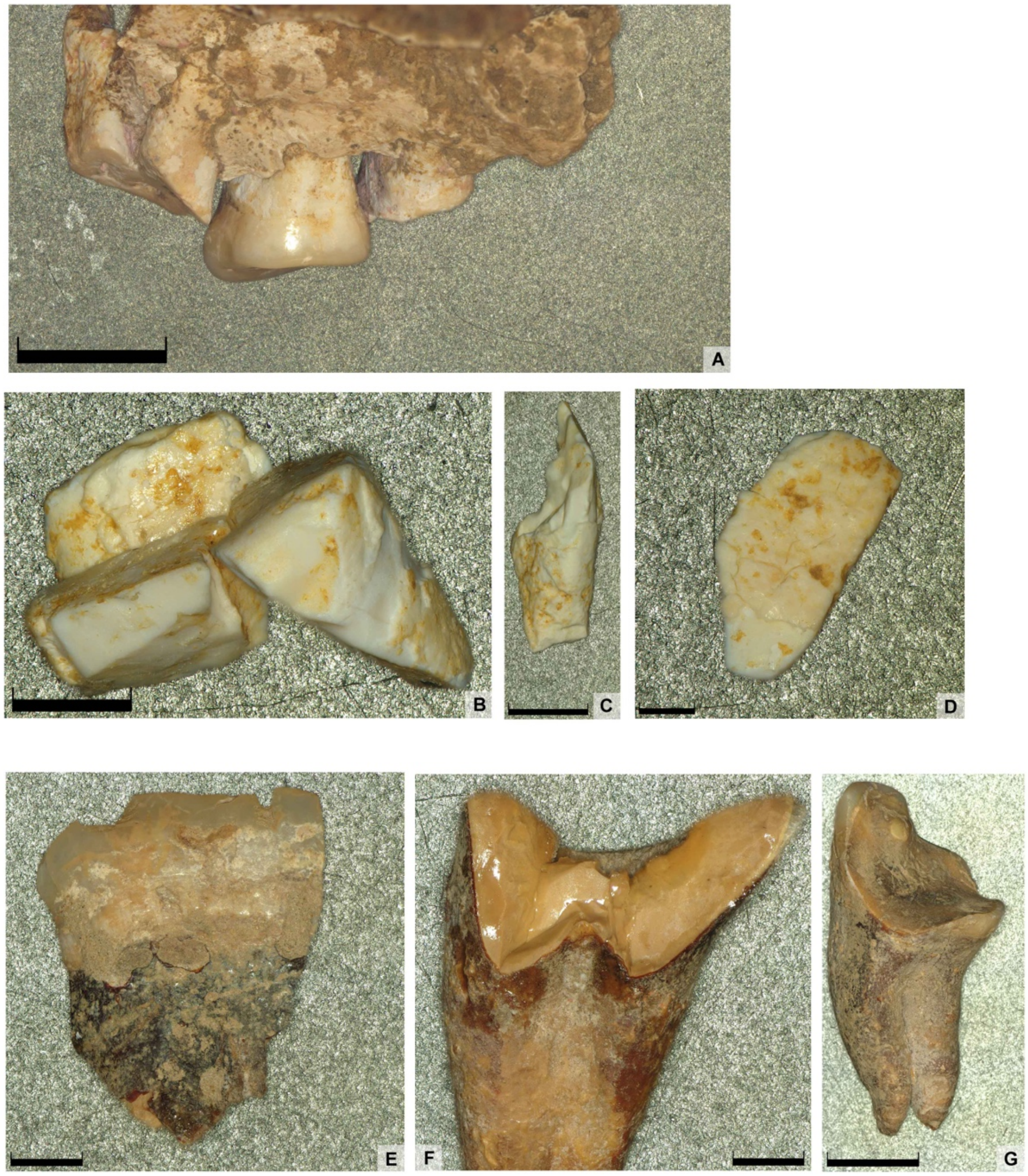

Fig. S10: Tooth samples used for biomolecular analysis. (A-D) Samples from the Late Pleistocene individual Maros-LBB-1a; (E-G) samples from the Middle Holocene individual CPL-R2a; (A) intact second molar (denoted Maros-LBB-1a-Gigi); (B-D) three tooth fragments (Maros-LBB-1a-Gigi1); (E-F) two fragments of an almost full tooth (denoted CPL-R2a-CAPP); (G) mandibular tooth (CPL-R2a-CAPP2) bearing circumferential dental wear. Scale bar in (A) is 10 mm; in (B, C, E) and (F) scale bars are 2 mm; in (D) and (G) they are 1 mm and 4 mm respectively.

Tables

Table S1: Tooth wear modification of CPL-R2a. R = right; L = left.

Teeth	Wear Type	Direction of wear	Aspect with greater degree of wear	Bilateral
R M^2<br>R M^1	Oblique plane wear	Bucco-lingual	Lingual	Unknown (left teeth damaged)
R M^2	Dental picking	Circumferential	Interproximal	No
R M^1	Circumferential	Circumferential	Buccal aspect (paracone)	No
R P^4<br>R P^3<br>R C^1	Circumferential	Circumferential	Across occlusal surfaces of P3 and canine	Yes
L P^4<br>L P^3<br>L C^1	Circumferential	Circumferential	Across occlusal surfaces of P3 and canine	Yes
R M_3<br>R M_2	Circumferential	Circumferential	Mediobuccal	Yes
R P_3	Circumferential	Circumferential	Cusps equally worn	Yes
R C_1	Plane	Unknown	Cusps equally worn	Yes
L I_1<br>L I_2	Plane	Medio-distal	Medial	Unknown (right teeth lost antemortem)

Table S2: Statistical analysis of data in Fig. S8.

One Way ANOVA Fig. S8
Data Table: Areca 12-22
Factor A: 8 Groups
WS, BS, WF, BF, WT, BT, arc 24hr, arc fresh

Analysis of Variance Results

Source	DF	SS	MS	F	P
Total	54	60.831248	1.1265046		
A	7	33.35048	4.7643543	8.1484132	< .0001
Error	47	27.480768	0.58469719		

IS	Intact *supari*
BS	Broken *supari*
IF	Intact 'fresh' areca
BF	Broken 'fresh' areca
IT	Intact *Areca triandra*
BT	Broken *A. triandra*
arc 24hr	10 µM arecoline incubated at 37˚ overnight
arc fresh	Fresh solution of 10 µM arecoline

Bonferroni's All Pairs Comparison

Comparison	Mean Difference	|t|	P	95% CL
IS vs BS	-1.46913	3.5944	0.0217	-2.8229 to -0.11535
IS vs IF	-1.33777	3.1446	0.0807	-2.7468 to 0.071293
IS vs BF	-2.32281	5.6831	< .0001	-3.6766 to -0.96902
IS vs IT	-2.38554	5.8365	< .0001	-3.7393 to -1.0318
IS vs BT	-1.82277	4.4597	0.0014	-3.1766 to -0.46898
IS vs arc 24hr	-2.36185	5.7786	< .0001	-3.7156 to -1.0081
IS vs arc fresh	-2.35606	5.7644	< .0001	-3.7099 to -1.0023
BS vs IF	0.131359	0.3088	1	-1.2777 to 1.5404
BS vs BF	-0.853677	2.0886	1	-2.2075 to 0.50011
BS vs IT	-0.91641	2.2421	0.8319	-2.2702 to 0.43738
BS vs BT	-0.353638	0.8652	1	-1.7074 to 1.0001
BS vs arc 24hr	-0.892713	2.1841	0.9513	-2.2465 to 0.46108
BS vs arc fresh	-0.88693	2.17	0.9827	-2.2407 to 0.46686
IF vs BF	-0.985036	2.3155	0.7	-2.3941 to 0.42403
IF vs IT	-1.04777	2.4629	0.49	-2.4568 to 0.3613
IF vs BT	-0.484997	1.1401	1	-1.8941 to 0.92407
IF vs arc 24hr	-1.02407	2.4072	0.5615	-2.4331 to 0.385
IF vs arc fresh	-1.01829	2.3936	0.5803	-2.4274 to 0.39078

BF vs IT	-0.062733	0.1535	1	-1.4165 to 1.2911
BF vs BT	0.500039	1.2234	1	-0.85375 to 1.8538
BF vs arc 24hr	-0.0390357	0.0955	1	-1.3928 to 1.3148
BF vs arc fresh	-0.0332529	0.0814	1	-1.387 to 1.3205
IT vs BT	0.562772	1.3769	1	-0.79102 to 1.9166
IT vs arc 24hr	0.0236973	0.058	1	-1.3301 to 1.3775
IT vs arc fresh	0.0294801	0.0721	1	-1.3243 to 1.3833
BT vs arc 24hr	-0.539074	1.3189	1	-1.8929 to 0.81471
BT vs arc fresh	-0.533292	1.3048	1	-1.8871 to 0.8205
arc 24hr vs arc fresh	0.00578285	0.0141	1	-1.348 to 1.3596

Controls after
One Way ANOVA Figure S3A
Data Table: Areca 12-22
Factor A: 8 Groups
IS, BS, IF, BF, IT, BT, arc 24hr, arc fresh

Analysis of Variance Results

Source	DF	SS	MS	F	P
Total	54	0.53106429	0.0098345238		
A	7	0.37076944	0.052967062	15.530455	< .0001
Error	47	0.16029485	0.0034105287		

Bonferroni's All Pairs Comparison

Comparison	Mean Difference	|t|	P	95% CL
IS vs BS	-0.20182	6.4653	< .0001	-0.30521 to -0.098426
IS vs IF	-0.160249	4.9322	0.0003	-0.26787 to -0.052633
IS vs BF	-0.208555	6.681	< .0001	-0.31195 to -0.10516
IS vs IT	-0.105659	3.3848	0.0405	-0.20905 to -0.0022647
IS vs BT	-0.191684	6.1406	< .0001	-0.29508 to -0.08829
IS vs arc 24hr	-0.0176393	0.5651	1	-0.12103 to 0.085755
IS vs arc fresh	-0.035923	1.1508	1	-0.13932 to 0.067471
BS vs IF	0.0415706	1.2795	1	-0.066045 to 0.14919
BS vs BF	-0.00673486	0.2158	1	-0.11013 to 0.096659
BS vs IT	0.0961611	3.0805	0.0966	-0.007233 to 0.19956
BS vs BT	0.0101363	0.3247	1	-0.093258 to 0.11353
BS vs arc 24hr	0.184181	5.9002	< .0001	0.080787 to 0.28757
BS vs arc fresh	0.165897	5.3145	< .0001	0.062503 to 0.26929
IF vs BF	-0.0483054	1.4868	1	-0.15592 to 0.059311
IF vs IT	0.0545906	1.6802	1	-0.053025 to 0.16221
IF vs BT	-0.0314343	0.9675	1	-0.13905 to 0.076182
IF vs arc 24hr	0.14261	4.3893	0.0018	0.034994 to 0.25023
IF vs arc fresh	0.124326	3.8265	0.0107	0.01671 to 0.23194
BF vs IT	0.102896	3.2963	0.0524	-0.00049817 to 0.20629

BF vs BT	0.0168711	0.5405	1	-0.086523 to 0.12027
BF vs arc 24hr	0.190916	6.116	< .0001	0.087521 to 0.29431
BF vs arc fresh	0.172632	5.5302	< .0001	0.069238 to 0.27603
IT vs BT	-0.0860249	2.7558	0.2326	-0.18942 to 0.017369
IT vs arc 24hr	0.0880196	2.8197	0.1964	-0.015375 to 0.19141
IT vs arc fresh	0.0697359	2.234	0.8478	-0.033658 to 0.17313
BT vs arc 24hr	0.174044	5.5755	< .0001	0.07065 to 0.27744
BT vs arc fresh	0.155761	4.9898	0.0002	0.052367 to 0.25915
arc 24hr vs arc fresh	-0.0182837	0.5857	1	-0.12168 to 0.08511

Table S3: Betel nut- and *P. betle*-related chemical compounds in the ancient human tooth specimens.

**Sample**	**Maros-LBB-1a-Gigi**	**Maros-LBB-1a-Gigi1**	**CPL-R2a-CAPP**	**CPL-R2a-CAPP2**
Betel nut (areca)				
Arecoline		√		√
Guvacoline				
Arecaidine				
Guvacine				
Oleic acid	√	√	√	√
Palmitic acid	√	√		
Arachidic acid	√	√		
Nicotine	√	√	√	√
Vanillin	√	√	√	√
Myristic acid	√	√		
Phenylalanine	√	√	√	√
Piper betle leaf				
Hydroxychavicol				
Eugenol	√	√	√	
Eucalyptol	√	√	√	
Anethole			√	
Citral	√	√	√	
Cumene	√	√	√	
Chavicol				
Acetyleugenol				
Methyeugenol				
Isosafrole				
Coumarin		√		
Monopalmitin		√	√	√
Stearic acid		√	√	√
Farnesene		√		√
Linolenic acid				
Oleic acid		√	√	√
Palmitic acid		√		√
Phytol		√		

Stigmasterol		√		
Malic acid			√	√
Hexadecanamide		√		

Table S4: Prehistoric evidence for betel nut chewing or staining in prehistoric human skeletal remains from Asia-Pacific.

Reference	Date	Location	Evidence	Deliberate or Habitual Staining	Chemical Analysis	Photos Available
**Mainland Southeast Asia**						
Pietrusewsky (1988) (*92*)	3500-1800 BP (Bronze/ Iron)	Non Pa Kluay, northeast Thailand	Possible staining (inconclusive)	N/A	No	No
Matsumura et al. (2001) (*93*)	2200-1700 BP (Iron Age)	Min Duc, northern Vietnam	Not reported by authors, but clear dark staining preserving the gum line can be seen in an image in the publication. M.V. examined this skull in person in 2024. All preserved teeth are stained, but on the labial surface only.	Deliberate	No	Yes
Oxenham et al. (2002) (*12*)	2400-1700 BP<br>(Iron Age)<br>Radiocarbon date only for later layers (1670 BP)	Nui Nap, northern Vietnam	Rich red-brown staining on labial surfaces of anterior teeth, SEM of etching, GC-MS	Deliberate	Yes. Some identical mass patterns between sample and standard. No arecoline	Yes

					identified. Tannins identified are not specific to *Areca* spp.	
Oxenham et al. (2002) (*12*)	~2480 BP[38] (Iron Age)	Gion Ca Vo, southern Vietnam	Rich red-brown staining on labial surfaces of anterior teeth	Deliberate	No	M.V. (pers. obs.) Image available (see Fig. S5).
Pietrusewsky and Ikehara-Quebral (2006) (*95*)	2200-1600 BP (Iron Age) Radiocarbon dated	Vat Komnou, Angkor Borei, Cambodia	Light brown to dark reddish-brown staining equally across all surfaces.	Habitual	No	No
Willis (2015) (*70*)	2150-1700 BP (Iron Age) Radiocarbon dated	Hoa Diem, central Vietnam	Labial surface stained, lingual surface is not. Dark reddish-brown stain on anterior teeth. Gum line preserved.	Deliberate	No	Yes
Willis (2015) (*70*)	3500-3000 BP (Neolithic) Radiocarbon dated	An Son, southern Vietnam	Staining on the posterior surfaces of teeth.<br>*Note:* M.V. has reviewed these teeth and believes the staining to be postmortem.	Habitual	No	No
Krais et al (2017) (*10*)	2550-2150 BP	Go O Chua, northern Vietnam	Reddish brown staining across all surfaces of dentition of one child	Deliberate	Yes	Yes

	(Iron Age) Radiocarbon dated		(except gum line), of anterior teeth of the adults			
Moonkham et al. (2025) (*13*)	3500-3000 BP (Neolithic) Layer radiocarbon dated to 4000-3000 BP, refined by relative dates and stratigraphic relationships.	Nong Ratchawat, central Thailand	None reported	Habitual	Yes. *Areca* spp. compounds identified including arecoline.	No
**Island Southeast Asia**						
Fox (1970) (*74*), Winters (1973) (*96*)	4630 BP (Pre-Neolithic), 2500-1500 BP (Metal Period) Pre-Neolithic is radiocarbon dated	Duyong Cave, Philippines	Metal Period- Teeth are stained reddish brown to black<br><br>Pre-Neolithic- "teeth stained"	N/A	No	No
Fox and Evangelista (1957) (*97*)	2000-1700 BP (Metal Period) Relative Dated	Bato Caves, Sorsogon Province, Philippines	Red staining on some teeth	N/A	No	No
Winters (1973) (*96*)	2500-1500 BP (Metal Period) Relative dated.	Uyaw Cave, Philippines	Teeth are stained reddish brown to black	N/A	No	No

Lien (1991) (*98*)	3300-2850 BP (Neolithic) (*99*), Radiocarbon dated	Peinan (Beinan), East Taiwan	'betel nut mastication'	N/A	No	No
Bellwood (1997), *primary source no longer available*: Bulbeck (1978) (*100*)	2000-1000 BP (Metal Period) Relative dated.	Lenag Buidane, Salebabu Island, Indonesia	Staining on teeth "consistent with betel nut"	N/A	No	No
Yankowski (2005) (*101*)	2500-1000 BP (Metal Period) Relative Dated	Bohol, Philippines	Reddish staining on a number of teeth	N/A	No	No
Bulbeck (2005) (*102*)	3250-1000 BP (Neolithic) Radiocarbon dated	Gua Cha, Malaysia	Pronounced staining on anterior teeth and premolars	N/A	No	No
Bulbeck (2005) (*72*), Shahidan et al. (2018) (*103*)	5700 BP (Pre-Neolithic) Radiocarbon date from a single shell without stratigraphic relationship to the other burials which may postdate the	Guar Kepah, Malaysia	Reddish staining on teeth and circumferential attrition. Just above the gum line. Severe periodontal disease. All teeth involved from incisors to third molars. Could	Habitual.	No	Yes

	mound. The burials are from three separate sites (Guar Kepah A,B, and C) Potshards discovered throughout the assemblages may suggest these are Neolithic or stratigraphic relationships are unreliable. Some Austronesian craniodental traits suggest post-contact is possible.		be differentiated from postmortem hematite light staining.			
Noerwidi (2012) (*104*)	3063 BP (Neolithic)<br>Radiocarbon dated	Song Keplek, Java, Indonesia	Reddish-brown stain on anterior labial teeth with exception of gum line. Lingual surface appears unstained. Severe periodontal disease.	Deliberate.	No	Yes
Pietrusewsky et al. (2013) (*76*), Pietrusewsky (2016) (*105*)	4800-4200 BP (Neolithic) Radiocarbon dating of cultural layer via ceramics.	Nankuanli East, Taiwan	Red-brown staining on all surfaces of teeth of most individuals, with severe circumferential attrition	Habitual	No	Yes

			patterns. Authors acknowledge that a proportion of the staining can be attributed to postmortem soil staining. Many bones are stained red from surrounding soil.			
Vlok (2016) (*106*)	2000-1500 BP (Metal Period)<br>Radiocarbon dated	Napa, Luzon, the Philippines	Reddish staining on teeth and circumferential attrition of molars. Severe periodontal disease, periapical lesion development, and severe dental calculus. TMJ degenerative joint disease.	Habitual	No	Yes
Koesbardiati and Murti (2019) (*78*)	~2990 BP (Neolithic)<br>Radiocarbon dated	Lewoleba, Nusa Tenggara, Indonesia	Reddish brown staining across all teeth. Severe attrition, periodontal disease and calculus.	Habitual	No	Yes

Koesbardiati and Murti (2019) (*78*)	3800-3300 BP (Neolithic) Radiocarbon dated	Liang Bua, Nusa Tenggara, Indonesia	Reddish brown staining across all teeth. Severe attrition, periodontal disease and calculus.	Habitual/ Also deliberate?	No	Yes
Samper Carro et al. (2022) (*71*)	~7500 BP (Pre-Neolithic) Radiocarbon dated	Tron Bon Lei, Alor Island, Indonesia	Reddish staining on buccal surfaces of teeth	N/A	No	No
M.V. (pers. obs.)	2022-1792 BP (Metal Period) Directly radiocarbon dated (*107*)	Nagsabaran, northern Luzon, Philippines	Reddish staining on the anterior labial surfaces of the incisors of a young male (Burial 4). Gum line preserved. Bones are also stained red, but teeth are white with the exception of the distinct areas of staining. Severe periodontal disease and calculus (but individual also had scurvy) (*108*)	Habitual	No	Yes (see Fig. S5)
**Pacific Islands**						
Leigh (1930) (*83*)	Latte Period (1000-500 BP)	Guam, Mariana	Staining of teeth orange to black	Deliberate and Habitual	No	No

	Relative dated	Islands, Micronesia	restricted to anterior teeth, premolars and firs molar, alveolar resorption			
Kirch et al (1989) (*80*)	Lapita (3600-1500 BP) Radiocarbon dated.	Mussau Islands, Bismarck Archipelago	Three loose teeth with staining. Only a canine appears to be completely discolored.	N/A	No	No
Hocart and Fankhauser (1996) (*58*)	Latte period (1000-500 BP) Relative dated.	Alaguan, Rota, Mariana Islands	Stained teeth on labial surfaces.	Deliberate	Yes. Presence of areca nut alkaloids.	No
Douglas et al. (1997) (*84*)	Latte period (1046-1665 BP) Radiocarbon dated.	Apurguan, Guam, Mariana Islands	Inferred due to low carious lesions.	N/A	No	No
Pietrusewsky et al. (2011) (*85*)	Latte period (1000-479 BP) Radiocarbon dated.	Rt 202, Tinian, Mariana Islands	Light to dark brown/ reddish staining	N/A	No	No
Pietrusewsky et al. (2014) (*86*)	Latte period (950-450 BP) Radiocarbon dated.	Saipan, Mariana Islands	Light to dark brown/ reddish staining	N/A	No	No
Fitzpatrick et al. (2003) (*81*); Stone et al. (2020) (*109*)	Pre-Latte Period (2850-2360 BP) Radiocarbon dated.	Chelechol ra Orrak, Palau	Reddish-black stains on teeth. TMJ degenerative joint disease.	Reported as habitual. However, richness in color (dark brown) is typically more consistent	Yes	No

				with findings of deliberate staining of scored teeth. Authors incorrectly score staining degree as evidence for degree of habitual use.		
Stone et al. (2017) (*82*)	Pre-Earthwork Period (3920-2540 BP)<br>Site has been radiocarbon dated, but most teeth appear to be from surface finds collected by tourists.	Ucheliungs Cave, Palau, Micronesia	“stained from habitual chewing”, “light occlusal wear”	Habitual	No	No

REFERENCES

1. R. J. Sullivan, E. H. Hagen, Psychotropic substance-seeking: Evolutionary pathology or adaptation? *Addiction* **97**, 389–400 (2002).

2. E. Guerra-Doce, Psychoactive substances in prehistoric times: Examining the archaeological evidence. *Time Mind* **8**, 91–112 (2015).

3. G. Samorini, The oldest archeological data evidencing the relationship of *Homo sapiens* with psychoactive plants: A worldwide overview. *J. Psychedelic Stud.* **3**, 63–80 (2019).

4. D. F. Rooney, *Betel Chewing Traditions in South-East Asia* (Oxford Univ. Press, 1993).

5. IARC Working Group on the Evaluation of Carcinogenic Risks to Humans, World Health Organisation (IARC Working Group on the Evaluation of Carcinogenic Risks to Humans), Betel-quid and areca-nut chewing and some areca-nut derived nitrosamines. *IARC Monogr. Eval. Carcinog. Risks Hum.* **85**, 1–334 (2004).

6. C. Stokes, J. A. Pino, D. W. Hagan, G. E. Torres, E. A. Phelps, N. A. Horenstein, R. L. Papke, Betel quid: New insights into an ancient addiction. *Addict. Biol.* **27**, e13223 (2022).

7. S. S. Strickland, Anthropological perspectives on use of the areca nut. *Addict. Biol.* **7**, 85–97 (2002).

8. T. J. Zumbroich, The origin and diffusion of betel chewing: A synthesis of evidence from South Asia, Southeast Asia and beyond. *EJ. Indian Med.* **1**, 63–116 (2008).

9. H. Rumgay, R. Shah, J. Vignat, O. Ayo-Yusuf, P. J. Chaturvedi, E. N. S. Guerra, P. C. Gupta, R. Gupta, S. Liu, C. Magnusson, M. Parascandola, Y. C. Paulino, N. Rezaei, K. Siddiqi, S. Warnakulasuriya, B. Lauby-Secretan, I. Soerjomataram, Global burden of oral cancer in 2022 attributable to smokeless tobacco and areca nut consumption: A population attributable fraction analysis. *Lancet Oncol.* **25**, 1413–1423 (2024).

10. S. Krais, M. Klima, L. M. Huppertz, V. Auwärter, M. J. Altenburger, M. A. Neukamm, Betel nut chewing in Iron Age Vietnam? Detection of *Areca catechu* alkaloids in dental enamel. *J. Psychoactive Drugs* **49**, 11–17 (2017).

11. A. A. Franke, X. Li, L. J. Custer, J. F. Lai, Chemical markers for short- and long-term areca nut exposure. *Subst. Use Misuse* **55**, 1395–1402 (2020).

12. M. F. Oxenham, C. Locher, N. L. Cuong, N. K. Thuy, Identification of *Areca catechu* (betel nut) residues on the dentitions of Bronze Age inhabitants of Nui Nap, northern Vietnam. *J. Archaeol. Sci.* **29**, 909–915 (2002).

13. P. Moonkham, S. Tushingham, M. Zimmermann, K. J. Brownstein, C. Devanwaropakorn, S. Duangsakul, D. R. Gang, Earliest direct evidence of bronze age betel nut use: Biomolecular analysis of dental calculus from Nong Ratchawat. Thailand. *Front. Environ. Archaeol.* **4**, 1622935 (2025).

14. P. Bellwood, *Prehistory of the Indo-Malaysian Archipelago* (ANU E-Press, 2007).

15. A. Brumm, D. Bulbeck, B. Hakim, B. Burhan, A. A. Oktaviana, I. Sumantri, Skeletal remains of a Pleistocene modern human (*Homo sapiens*) from Sulawesi. *PLOS ONE* **16**, e0257273 (2021).

16. B. Burhan, B. Hakim, I. Sumantri, Suryatman, A. M. Saiful, A. A. Oktaviana, R. Sardi, Hasliana, M. Ramli, L. Siagian, A. Jusdi, Abdullah, F. A. Syahdar, Hamrullah, I. Ilyas, P. H. Muhammad, S. S. Budi, N. I. Djindar, S. Adhityatama, R. Lebe, M. N. R. Ririmasse, I. Mahmud, A. Duli, Y. L. Perston, M. W. Moore, M. Sontag-González, B. Li, G. D. van den Bergh, M. Aubert, R. Grün, D. P. McGahan, M. C. Langley, E. C. James, T. Manne, I. Moffat, B. Jones, A. Brumm, A near-continuous archaeological record of Pleistocene human occupation at Leang Bulu Bettue, Sulawesi, Indonesia. *PLOS ONE* **20**, e0337993 (2025).

17. Suryatman, Fakhri, B. Hakim, Y. Perston, R. Sardi, K. Newman, M. N. Hasanuddin, K. Muda, Incised stone artefact in the context of Middle Holocene burials at Cappalombo 1, South Sulawesi, Indonesia. *SPAFA J.* **5**, 684 (2021).

18. D. Bulbeck, M. Pasqua, A. Di Lello, Culture history of the Toalean of South Sulawesi. Indonesia. *Asian Perspect.* **39**, 71–108 (2000).

19. S. Carlhoff, A. Duli, K. Nägele, M. Nur, L. Skov, I. Sumantri, A. A. Oktaviana, B. Hakim, B. Burhan, F. A. Syahdar, D. P. McGahan, D. Bulbeck, Y. L. Perston, K. Newman, A. M. Saiful, M. Ririmasse, S. Chia, D. A. T. Hasanuddin, S. Pulubuhu, C. Supriadi, B. M. Jeong, K. Peter, A. Prüfer, J. Powell, C. Krause, A. B. Posth, A. Brumm, Genome of a middle Holocene hunter-gatherer from Wallacea. *Nature* **596**, 543–547 (2021).

20. D. H. Ubelaker, Pipe wear: Dental impact of colonial American culture. *Anthropologie* **34**, 321–327 (1996).

21. S. M. Alaydrus, M. D. Artaria, Y.-F. Chen, The impact of chewing betel nuts on human dentition in Indonesia: A literature review. *Maj. Ked. Gi.* **56**, 273–279 (2023).

22. S. Mondal, R. Biswas, S. Basu, M. Chowdhury, *Areca triandra* Roxb. ex Buch.-Ham. (Arecaceae): New record for West Bengal, India. *Plant Arch.* **18**, 1700–1702 (2018).

23. J. Chmiel, A. Malinowska, The neural correlates of chewing gum-A neuroimaging review of its effects on brain activity. *Brain Sci.* **15**, 657 (2025).

24. N. A. Horenstein, M. Quadri, C. Stokes, M. Shoaib, R. L. Papke, Cracking the betel nut: Cholinergic activity of Areca alkaloids and related compounds. *Nic. Tob. Res.* **21**, 805–812 (2017).

25. P. G. Osborne, R. R. Pasupuleti, M.-T. Wu, C.-H. Lee, V. K. Ponnusamy, LC-MS/MS measurement of alkaloids in alkaline extracts of Areca nut preparations and their physiological effects. *Process Biochem.* **118**, 65–73 (2022).

26. F. K. Lichtenberk, Did speakers of Proto Oceanic chew betel? *J. Polyn. Soc.* **107**, 335–363 (1998).

27. V. Jain, A. Garg, M. Parascandola, P. Chaturvedi, S. S. Khariwala, I. Stepanov, Analysis of alkaloids in areca nut-containing products by liquid chromatography-tandem mass spectrometry. *J. Agric. Food Chem.* **65**, 1977–1983 (2017).

28. S. A. Norton, Betel: Consumption and consequences. *J. Am. Acad. Dermatol.* **38**, 81–88 (1998).

29. Q. Y. Chen, Y. Zhang, Y. Ma, M. Zhuo, Inhibition of cortical synaptic transmission, behavioral nociceptive, and anxiodepressive-like responses by arecoline in adult mice. *Mol. Brain* **17**, 39 (2024).

30. T. R. Maloney, I. E. Dilkes-Hall, M. Vlok, A. A. Oktaviana, P. Setiawan, A. A. D. Priyatno, M. Ririmasse, I. M. Geria, M. A. R. Effendy, B. Istiawan, F. T. Atmoko, S. Adhityatama, I. Moffat, R. Joannes-Boyau, A. Brumm, M. Aubert, Surgical amputation of a limb 31,000 years ago in Borneo. *Nature* **609**, 547–551 (2022).

31. A. Brumm, M. C. Langley, M. W. Moore, B. Hakim, M. Ramli, I. Sumantri, B. Burhan, A. M. Saiful, L. Siagian, Suryatman, R. Sardi, A. Jusdi, Abdullah, A. P. Mubarak, Hasliana, Hasrianti, A. A. Oktaviana, S. Adhityatama, G. D. van den Bergh, M. Aubert, J.-X. Zhao, J. Huntley, B. Li, R. G. Roberts, E. W. Saptomo, Y. Perston, R. Grün, Early human symbolic behavior in the Late Pleistocene of Wallacea. *Proc. Natl. Acad. Sci. U.S.A.* **114**, 4105–4110 (2017).

32. A. Brumm, M. Langley, B. Hakim, Y. Perston, Suryatman, A. A. Oktaviana, B. Burhan, M. W. Moore, Scratching the surface: Engraved cortex as portable art in Pleistocene Sulawesi. *J. Archaeol. Method Theory* **27**, 670–698 (2020).

33. M. C. Langley, B. Hakim, A. A. Oktaviana, B. Burhan, I. Sumantri, P. H. Sulistyarto, R. Lebe, D. McGahan, A. Brumm, Portable art from Pleistocene Sulawesi. *Nat. Hum. Behav.* **4**, 597–602 (2020).

34. Y. L. Perston, M. W. Moore, B. Suryatman, B. Burhan, A. A. Hakim, R. L. Oktaviana, A. B. Hasliana, A. Brumm, Stone-flaking technology at Leang Bulu Bettue, South Sulawesi, Indonesia. *Archaeol. Oceania* **57**, 249–272 (2022).

35. Fakhri, "Eksplorasi gua-gua prasejarah kawasan kars Bontocani: Fase hunian dan lapisan budaya dari Pleistosen akhir hingga Holosen di dataran tinggi Sulawesi Selatan" (Balai Arkeologi Sulawesi Selatan, 2018).

36. Y. L. Perston, M. Moore, Suryatman, M. Langley, B. Hakim, A. A. Oktaviana, A. Brumm, A standardised classification scheme for the Mid-Holocene Toalean artefacts of South Sulawesi, Indonesia. *PLOS ONE* **16**, e0251138 (2021).

37. Suryatman, "Perbandingan teknologi artefak batu Toalean berdasarkan ketinggian kawasan situs di Sulawesi Selatan," thesis, University of Hasanuddin, Makassar, Indonesia (2021).

38. C. B. Ramsey, Methods for summarizing radiocarbon datasets. *Radiocarbon* **59**, 1809–1833 (2017).

39. P. J. Reimer, E. Bard, A. Bayliss, J. W. Beck, P. G. Blackwell, C. B. Ramsey, C. E. Buck, H. Cheng, R. L. Edwards, M. Friedrich, P. M. Grootes, T. P. Guilderson, H. Haflidason, I. Hajdas, C. Hatté, T. J. Heaton, D. L. Hoffmann, A. G. Hogg, K. A. Hughen, K. F. Kaiser, B. Kromer, S. W. Manning, M. Niu, R. W. Reimer, D. A. Richards, E. M. Scott, J. R. Southon, R. A. Staff, C. S. M. Turney, J. van der Plicht, IntCal13 and Marine13 radiocarbon age calibration curves 0–50,000 years cal BP. *Radiocarbon* **55**, 1869–1887 (2013).

40. C. B. Ramsey, OxCal v.4.4.4 [software] (2021); https://c14.arch.ox.ac.uk/oxcal.html.

41. P. J. Reimer, W. E. N. Austin, E. Bard, A. Bayliss, P. G. Blackwell, C. B. Ramsey, M. Butzin, H. Cheng, R. L. Edwards, M. Friedrich, P. M. Grootes, T. P. Guilderson, I. Hajdas, T. J. Heaton, A. G. Hogg, K. A. Hughen, B. Kromer, S. W. Manning, R. Muscheler, J. G. Palmer, C. Pearson, J. van der Plicht, R. W. Reimer, D. A. Richards, E. M. Scott, J. R. Southon, C. S. M. Turney, L. Wacker, F. Adolphi, U. Büntgen, M. Capano, S. M. Fahrni, A. Fogtmann-Schulz, R. Friedrich, P. Köhler, S. Kudsk, F. Miyake, J. Olsen, F. Reinig, M. Sakamoto, A. Sookdeo, S. Talamo, The IntCal20 northern hemisphere radiocarbon age calibration curve (0–55 cal kBP). *Radiocarbon* **62**, 725–757 (2020).

42. N. S. Chu, Effects of betel chewing on the central and autonomic nervous systems. *J. Biomed. Sci.* **8**, 229–236 (2001).

43. P. F. Liu, Y. F. Chang, The controversial roles of areca nut: Medicine or toxin? *Int. J. Mol. Sci.* **24**, 8996 (2023).

44. R. L. Papke, C. Stokes, Working with OpusXpress: Methods for high volume oocyte experiments. *Methods* **51**, 121–133 (2010).

45. A. Das, S. Giri, A review on role of arecoline and its metabolites in the molecular pathogenesis of oral lesions with an insight into current status of its metabolomics. *Prague Med. Rep.* **121**, 209–235 (2020).

46. A. Ansari, T. Mahmood, P. Bagga, F. Ahsan, A. Shamim, S. Ahmad, M. Shariq, S. Parveen, *Areca catechu*: A phytopharmacological legwork. *Food Front.* **2**, 163–183 (2021).

47. Y. Yang, H. Huang, Z. Cui, J. Chu, G. Du, UPLC–MS/MS and network pharmacology-based analysis of bioactive anti-depression compounds in betel nut. *Drug Des. Devel. Ther.* **15**, 4827–4836 (2021).

48. K. Ghosh, T. K. Bhattacharya, Chemical constituents of *Piper betle* Linn. (Piperaceae) roots. *Molecules* **10**, 798–802 (2005).

49. P. Biswas, U. Anand, S. C. Saha, N. Kant, T. Mishra, H. Masih, A. Bar, D. K. Pandey, N. K. Jha, M. Majumder, N. Das, V. S. Gadekar, M. S. Shekhawat, M. Kumar, Radha, J. Proćków, J. M. P. de la Lastra, A. Dey, Betel vine (*Piper betle* L.): A comprehensive insight into its ethnopharmacology, phytochemistry, and pharmacological, biomedical and therapeutic attributes. *J. Cell. Mol. Med.* **26**, 3083–3119 (2022).

50. R. K. Gupta, P. Guha, P. P. Srivastav, Phytochemical and biological studies of betel leaf (*Piper betle* L.): Review on paradigm and its potential benefits in human health. *Acta Ecol. Sin.* **43**, 721–732 (2023).

51. T. Singh, P. Singh, V. K. Pandey, R. Singh, A. H. Dar, A literature review on bioactive properties of betel leaf (*Piper betel* L.) and its applications in food industry. *Food Chem. Adv.* **3**, 100536 (2023).

52. L. T. T. Nguyen, T. T. Nguyen, H. N. Nguyen, Q. T. P. Bui, Simultaneous determination of active compounds in *Piper betle* Linn. leaf extract and effect of extracting solvents on bioactivity. *Eng. Rep.* **2**, e12246 (2020).

53. N. I. Azahar, N. M. Mokhtar, M. A. Arifin, Piper betle: A review on its bioactive compounds, pharmacological properties, and extraction process. *IOP Conf. Ser. Mater. Sci. Eng.* **991**, 012044 (2020).

54. C. Cattaneo, F. Gigli, F. Lodil, M. Grandi, The detection of morphine and codeine in human teeth: An aid in the identification and study of human skeletal remains. *J. Forensic Odontostomatol.* **21**, 1–5 (2003).

55. G. Ottaviani, R. Cameriere, M. Cippitelli, R. Froldi, G. Tassoni, M. Zampi, M. Cingolani, Determination of drugs of abuse in a single sample of human teeth by a gas chromatography–mass spectrometry method. *J. Anal. Toxicol.* **41**, 32–36 (2017).

56. I. Bianchi, M. Cippitelli, E. Buratti, A. Cerioni, G. Mietti, M. Focardi, R. Grifoni, R. Scendoni, R. Froldi, M. Cingolani, V. Pinchi, A pilot study on post-mortem determination of drug abuse on dental tissues. *Forensic Sci. Int.* **363**, 112185 (2024).

57. M. Klima, M. J. Altenburger, J. Kempf, V. Auwärter, M. A. Neukamma, Determination of medicinal and illicit drugs in post mortem dental hard tissues and comparison with analytical results for body fluids and hair samples. *Forensic Sci. Int.* **265**, 166–171 (2016).

58. C. H. Hocart, B. Fankhauser, Betel nut residues in archaeological samples of human teeth from the Mariana Islands. *Experientia* **52**, 281–285 (1996).

59. J. W. Eerkens, Dental calculus as a source of ancient alkaloids: Detection of nicotine by LCMS in calculus samples from the Americas. *J. Archaeol. Sci. Rep.* **18**, 509–515 (2018).

60. T. D. White, M. T. Black, P. A. Folkens, *Human Osteology* (Academic Press, 2012).

61. D. E. Walrath, P. Turner, J. Bruzek, Reliability test of the visual assessment of cranial traits for sex determination. *Am. J. Phys. Anthropol.* **125**, 132–137 (2004).

62. S. Hillson, *Dental Anthropology* (Cambridge Univ. Press, 2023).

63. D. K. Latinis, The development of subsistence system models for Island Southeast Asia and Near Oceania: The nature and role of arboriculture and arboreal-based economies. *World Archaeol.* **32**, 41–67 (2000).

64. I. C. Glover, Prehistoric plant remains from Southeast Asia, with special reference to rice. *S. Asian Archaeol.* **7**, 7–37 (1977).

65. N. V. Oliveira, "Returning to East Timor: Prospects and possibilities from an archaeobotanical project in the new country," in *Uncovering Southeast Asia's Past: Selected Papers from the 10th International Conference of the European Association of Southeast Asian Archaeologists* (National University of Singapore Press, 2006), pp. 88–97.

66. C. F. Gorman, Excavations at Spirit Cave, north Thailand: Some interim interpretations. *Asian Perspect.* **13**, 79–107 (1970).

67. D. E. Yen, "Hoabinhian horticulture? The evidence and the questions from northwest Thailand," in *Sunda and Sahul: Prehistoric Studies in Southeast Asia, Melanesia and Australia* (Academic Press, 1977), pp. 567–599.

68. S. Williams, A. Malik, S. Chowdhury, S. Chauhan, Sociocultural aspects of areca nut use. *Addict. Biol.* **7**, 147–154 (2002).

69. T. J. Zumbroich, The ethnobotany of teeth blackening in Southeast Asia. *Ethnobot. Res. Appl.* **7**, 381–398 (2009).

70. A. C. J. Willis, "The bioarchaeology of An Sơn and Hòa Diêm: Biosocial insights into prehistoric southern Vietnam," thesis, The Australian National University, Canberra, Australia (2015).

71. S. C. Samper-Carro, S. O'Connor, Mahirta, S. Kealy, C. Shipton, Talking Dead. New burials from Tron Bon Lei (Alor Island, Indonesia) inform on the evolution of mortuary practices from the terminal Pleistocene to the Holocene in Southeast Asia. *PLOS ONE* **17**, e0267635 (2022).

72. F. Bulbeck, "The Guar Kepah human remains," in *The Perak Man and Other Prehistoric Skeletons of Malaysia* (Penerbit Universiti Sains Malaysia, 2005), pp. 383–423.

73. M. C. Swete Kelly, "Early pottery in Island Southeast Asia," in *Handbook of East and Southeast Asian Archaeology* (Springer, 2017), pp. 397–418.

74. R. B. Fox, *The Tabon Caves: Archaeological Explorations and Excavations on Palawan Island, Philippines* (National Museum of the Philippines, 1970).

75. K. Wiradnyana, Hoabinhian and Austronesia: the root of diversity in the western part of Indonesia. *Eur. Sci. J.* **12**, 131 (2016).

76. M. Pietrusewsky, A. Lauer, M. T. Douglas, C.-H. Tsang, K.-T. Li, Dental indicators of health in early Neolithic and Iron Age skeletons from Taiwan. *J. Austron. Stud.* **4**, 1–34 (2013).

77. K. Li, Identification of Pomadasys species (Pisces, Haemulidae) from an archaeological midden site in Nankuanli East (Taiwan), based on otolith morphology. *Raffles Bull. Zool.* **61**, 293–302 (2013).

78. T. Koesbardiati, D. B. Murti, Betel quid consumption and dental pathology at East Nusa Tenggara's Lewoleba and Liang Bua prehistoric communities. *Berkala Arkeologi* **39**, 121–138 (2019).

79. M. F. Oxenham, Dental health in Northern Vietnam: Pre Metal through Early Metal Periods. *IPPA Bull.* **22**, 121–134 (2002).

80. P. V. Kirch, D. R. Swindler, C. G. Turner, Human skeletal and dental remains from Lapita sites (1600–500 BC) in the Mussau Islands, Melanesia. *Am. J. Phys. Anthropol.* **79**, 63–76 (1989).

81. S. M. Fitzpatrick, G. C. Nelson, R. Reeves, The prehistoric chewing of betel nut (*Areca catechu*) in western Micronesia. *People Cult. Ocean.* **19**, 55–65 (2003).

82. J. H. Stone, S. M. Fitzpatrick, M. F. Napolitano, Disproving claims for small-bodied humans in the Palauan archipelago. *Antiquity* **91**, 1546–1560 (2017).

83. R. W. Leigh, Dental morphology and pathology of prehistoric Guam. *J. Dent. Res.* **10**, 451–479 (1930).

84. M. T. Douglas, M. Pietrusewsky, R. M. Ikehara-Quebral, Skeletal biology of Apurguan: A precontact Chamorro site on Guam. *Am. J. Phys. Anthropol.* **104**, 291–313 (1997).

85. M. Pietrusewsky, M. T. Douglas, M. K. Swift, R. A. Harper, M. A. Fleming, An assessment of health and lifestyle among prehistoric Chamorro from Tinian Island in the northern Mariana Islands. *Am. J. Phys. Anthropol.* **52**, 238–239 (2011).

86. M. Pietrusewsky, M. T. Douglas, M. K. Swift, R. A. Harper, M. A. Fleming, Health in ancient Mariana Islanders: A bioarchaeological perspective. *J. Isl. Coast. Archaeol.* **9**, 319–340 (2014).

87. C. Higham, The bronze age of Southeast Asia: New insight on social change from Ban Non Wat. *Camb. Archaeol. J.* **21**, 365–389 (2011).

88. C. Higham, A. Kijngam, *The Excavation of Ban Non Wat: The Bronze Age* (Thai Fine Arts Department, 2012).

89. C. Higham, A. Kijngam, *The Origins of the Civilization of Angkor*, *Volume 4: The Excavation of Ban Non Wat. Part II: The Neolithic Occupation* (Fine Arts Department of Thailand, 2011).

90. N. Tayles, "The excavation of Khok Phanom Di, a prehistoric site in Central Thailand, Volume 5: The people" (Research Committee Report, Research Committee Society of Antiquaries London, 1999).

91. M. F. Oxenham, H. Matsumura, N. Kim Dung, *Man Bac: The Excavation of a Neolithic Site in Northern Vietnam, The Biology* (Terra Australis 33, ANU ePress, 2011).

92. M. Pietrusewsky, *Prehistoric Human Remains from Non Pa Kluay, Vol. 17 Northeast Thailand* (University of Otago Studies in Prehistoric Anthropology, Department of Anthropology, University of Otago, 1988).

93. H. Matsumura, L. C. Nguyen, K. T. Nguyen, T. Anezake, Dental morphology of the early Hoabinian, the Neolithic Da But and the Metal Age Dong Son civilized peoples in Vietnam. *Z. Morphol. Anthropol.* **83**, 59–73 (2001).

94. K. S. Nguyen, Vietnam's Middle and Late Neolithic marine cultures. *J. Soc. Sci. Humanit.* **64**, 112–128 (2022).

95. M. Pietrusewsky, R. Ikehara-Quebral, The bioarchaeology of the Vat Komnou cemetery, Angkor Borei, Cambodia. *Bull. Indo-Pacific Prehist. Assoc.* **26**, 86–97 (2006).

96. N. J. Winters, An application of dental anthropological analysis to the human dentition of two Early Metal Age sites, Palawan, Philippines. *Asian Perspect.* **17**, 28–35 (1974).

97. R. B. Fox, A. Evangelista, The Bato Caves, Sorsogon Province, Philippines. *Univ. Manila J. East Asiatic Stud.* **6**, 49–56 (1957).

98. C.-M. Lien, The Neolithic archaeology of Taiwan and the Peinan excavations. *Bull. Indo-Pacific Prehist. Assoc.* **11**, 339–352 (1991).

99. R. Pearson, "Taiwan and its place in East Asian prehistory," in *Anthropological Studies of the Taiwan Area: Accomplishments and Prospects* (Department of Anthropology, National Taiwan University, 1989), pp. 111–141.

100. F. Bulbeck, "Analysis of a skeletal assemblage from Leang Buidane, Talaud Islands, Indonesia," thesis, The Australian National University, Canberra, Australia (1978).

101. A. Yankowski, "Trade, technologies & traditions: The analysis of artifacts recovered from a metal age burial site in district Ubujan, Tagbilaran city, Bohol, Central Philippines," thesis, San Francisco State University, San Francisco, US (2005).

102. F. Bulbeck, "The Gua Cha burials," in *The Perak Man and Other Prehistoric Skeletons of Malaysia* (Penerbit Universiti Sains Malaysia, 2005), pp. 253–309.

103. S. Shahidan, N. A. M. I. Mokhtar Saidin, N. K. Talib, S. Shyeh, Tapak Guar Kepah: Bukti perkuburan masyarakat Neolitik di dalam Timbunan Cangkerang Laut. *Melayu: Jurnal Antarabangsa Dunia Melayu* **11**, 239–244 (2018).

104. S. Noerwidi, Rekonstruksi aspek biologis dan konteks budaya rangka manusia Holosen, Song Keplek 5. *Berkala Arkeologi* **32**, 135–150 (2012).

105. M. Pietrusewsky, A. Lauer, M. T. Douglas, C.-H. Tsang, K.-T. Li, Patterns of health in Early Neolithic and Iron Age Taiwan. *Anthropol. Sci.* **124**, 117–133 (2016).

106. M. Vlok, "Healing bones: A case study of healthcare practice in the Metal Period, Philippines," thesis, The Australian National University, Canberra, Australia (2016).

107. M. Oxenham, A. Willis, H.-C. Hung, R. Page, H. Matsumura, "Dealing with death in late Neolithic to Metal period Nagsabaran, the Philippines," in *The Routledge Handbook of Bioarchaeology in Southeast Asia and the Pacific Islands* (Routledge, 2015), pp. 339–366.

108. C. Boucher, D. Armelle, J. J. Miszkiewicz, M. J. L. A. Bolunia, A. S. De Leon, A. L. Peñalosa, P. S. Pagulayan, A. V. Soranio, M. Oxenham, M. Vlok, Nutritional deficiency contributing to physical impairment of an individual in the Metal period Philippines (∼2000–1800 BP). *Int. J. Osteoarchaeol.* **36**, 221–230 (2026).

109. J. H. Stone, G. C. Nelson, S. M. Fitzpatrick, Temporomandibular joint osteoarthritis at Chelechol ra Orrak, Palau. *Int. J. Paleopathol.* **28**, 20–31 (2020).
